# Tuning infrared plasmon resonances in doped metal-oxide nanocrystals through cation-exchange reactions

**DOI:** 10.1038/s41467-019-09165-2

**Published:** 2019-03-27

**Authors:** Zeke Liu, Yaxu Zhong, Ibrahim Shafei, Ryan Borman, Soojin Jeong, Jun Chen, Yaroslav Losovyj, Xinfeng Gao, Na Li, Yaping Du, Erik Sarnello, Tao Li, Dong Su, Wanli Ma, Xingchen Ye

**Affiliations:** 1 Jiangsu Key Laboratory for Carbon-Based Functional Materials & Devices, Institute of Functional Nano & Soft Materials (FUNSOM), Joint International Research Laboratory of Carbon-Based Functional Materials and Devices, Soochow University, 199 Ren’ai Road, 215123 Suzhou, Jiangsu China; 2 Department of Chemistry, Indiana University, 800 East Kirkwood Avenue, Bloomington, IN 47405 USA; 3 Center for Functional Nanomaterials, Brookhaven National Laboratory, Upton, NY 11973 USA; 4 Frontier Institute of Science and Technology jointly with College of Science, Xi’an Jiaotong University, 710054 Xi’an, Shanxi Province China; 5 School of Materials Science and Engineering & National Institute for Advanced Materials, Nankai University, 300350 Tianjin, China; 6 Department of Chemistry and Biochemistry, Northern Illinois University, 1425 W. Lincoln Hwy., DeKalb, IL 60115 USA; 7 X-ray Science Division, Argonne National Laboratory, 9700 South Cass Avenue, Lemont, IL 60439 USA

## Abstract

Metal-oxide nanocrystals doped with aliovalent atoms can exhibit tunable infrared localized surface plasmon resonances (LSPRs). Yet, the range of dopant types and concentrations remains limited for many metal-oxide hosts, largely because of the difficulty in establishing reaction kinetics that favors dopant incorporation by using the co-thermolysis method. Here we develop cation-exchange reactions to introduce p-type dopants (Cu^+^, Ag^+^, etc.) into n-type metal-oxide nanocrystals, producing programmable LSPR redshifts due to dopant compensation. We further demonstrate that enhanced n-type doping can be realized via sequential cation-exchange reactions mediated by the Cu^+^ ions. Cation-exchange transformations add a new dimension to the design of plasmonic nanocrystals, allowing preformed nanocrystals to be used as templates to create compositionally diverse nanocrystals with well-defined LSPR characteristics. The ability to tailor the doping profile postsynthetically opens the door to a multitude of opportunities to deepen our understanding of the relationship between local structure and LSPR properties.

## Abstract (web summary)

Doping semiconductor nanocrystals with impurity atoms is a key pathway for tuning their plasmonic properties. Here, the authors use a cation exchange strategy to dope p-type or n-type metal ions into n-type metal-oxide nanocrystals, post-synthetically tailoring their localized surface plasmon resonances in the infrared region.

## Introduction

Doping, the intentional introduction of impurity atoms into a host lattice, is an essential process for developing semiconductor materials and devices. Over the past three decades, researchers have been studying how foreign atoms can be controllably incorporated into semiconductor nanocrystals (NCs) in order to tailor their optical and electronic properties^1–5^. In bulk semiconductors, substitutional dopant atoms with extra valence electrons can introduce shallow donor levels below the conduction band and when thermally ionized can produce n-type semiconductors that contain excess electrons. These electrons (or holes for p-type doping) are available as mobile charge carriers responsible for current transport. In semiconductor NCs, the collective oscillation of confined charge carriers in response to incident radiation can lead to the so-called localized surface plasmon resonance (LSPR)^6–10^.

The field of plasmonics can benefit greatly from the development of new nanomaterials with tunable carrier concentration and carrier dynamics^6–8,11,12^. Distinct from conventional metal-based plasmonic materials for which electron density is largely fixed and postsynthetic variation is typically not possible, the carrier density and thereby the LSPR energies of semiconductor NCs made of metal oxides^13–22^, chalcogenides^9,23–27^, phosphides^28,29^, nitrides^30^, and silicon^31^ can be synthetically adjusted to cover the near-infrared (NIR) and the mid-infrared regions. The unique plasmonic properties of semiconductor NCs are being harnessed for new applications and technologies including but not limited to surface-enhanced infrared absorption spectroscopy (SEIRAS)^32,33^, smart windows^10^, low-loss optical metamaterials^11,34^, and bioimaging^23,35,36^. During the past 5 years, impressive progress has been made toward the syntheses of various plasmonic metal-oxide NCs, such as tin-doped In_2_O_3_ (ITO)^13,22,37^, indium-doped CdO (ICO)^16,17^, aluminum-doped ZnO (AZO) NCs^14,38^, to name a few. Colloidal synthesis of doped metal-oxide NCs generally involves co-thermolysis of mixed metal precursors, which requires fine-tuning the relative chemical reactivity of precursors to establish reaction kinetics that favors dopant incorporation^6,39^. The presence of dopant ions in high concentrations can often significantly alter the nucleation and growth kinetics of host NCs, resulting in the loss of NC size and shape control or unsuccessful doping^16,40^. A more fundamental issue associated with the co-thermolysis method is the lack of control of the spatial distribution of dopants, which has recently been shown to play a vital role in determining the plasmonic response of metal-oxide NCs^41^. Alternatively, chemical transformation of pre-synthesized metal-oxide NCs with well-defined structural and spectral properties into derivative NCs could provide improved, simultaneous control of the chemical identity and concentration profile of dopants. Among different “conversion chemistry” strategies, cation-exchange transformations have been extensively studied in various semiconductor NCs for preparation of metastable or doped NCs^42–45^. However, to the best of our knowledge, cation-exchange has not been exploited in doped metal-oxide NCs to deliberately vary the carrier concentration and thus the LSPR frequency.

In this report, we describe the use of cation-exchange reactions to tailor the elemental composition and the resulting LSPR characteristics of doped metal-oxide NCs. We demonstrate that several p-type impurities, which have been difficult to incorporate by using the conventional co-thermolysis method, can be doped into various n-type metal-oxide NCs giving rise to predictable LSPR peak redshifts. We further show that rationally designed sequential cation-exchange reactions can lead to enhanced n-type doping. Collectively, our work demonstrates that cation-exchange is an important synthetic tool for creating novel plasmonic metal-oxide NCs via postsynthetic modification of NC composition independently of NC’s size, shape, and crystal structure.

## Results

### Synthesis of Cu:ICO NCs via cation-exchange reactions

ICO and ITO NCs were chosen as model systems because they can support an intense infrared LSPR whose peak wavelength and linewidth are tunable by controlling the concentration of different substitutional dopants^13,16,22^. This LSPR feature provides a simple optical probe for monitoring reaction progression and for gaining mechanistic understanding of cation-exchange reactions on metal-oxide NCs.

**Fig. 1:**
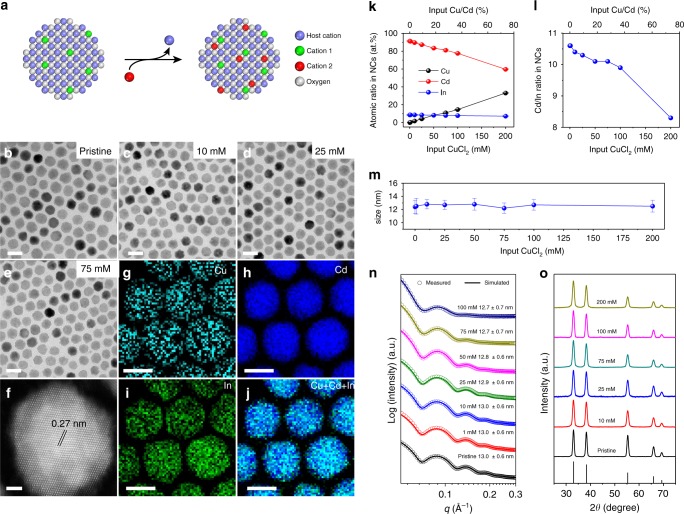
Synthesis and structural characterization of Cu:ICO NCs. **a** Reaction scheme of the selective cation-exchange reaction. **b**–**e** TEM images of (**b**) ICO NCs (*λ*_initial_ = 2196 nm) and **c**–**e** Cu:ICO NCs synthesized by reacting ICO NCs with varying concentrations of CuCl_2_. **f** STEM-HAADF image of a single NC for samples shown in (**e**). **g**–**j** STEM-EELS elemental maps of Cu:ICO NCs obtained after reacting ICO NCs with 75 mM CuCl_2_. Elemental maps for (**g**) Cu, (**h**) Cd, (**i**) In, and (**j**) overlay of three micrographs are shown. Scale bars: (**b**–**e**) 20 nm, (**f**) 2 nm, and (**g**–**j**) 10 nm. **k** Elemental composition of ICO and Cu:ICO NCs measured with SEM-EDS (assuming Cu + Cd + In = 100%). The total Cd content of pristine ICO NCs was determined by using ICP-MS. **l** Plot of the atomic ratio between Cd and In for Cu:ICO NCs vs. the input Cu/Cd ratio. **m** and **n** Statistical analysis of NC size for ICO and Cu:ICO NCs based on (**m**) TEM images and (**n**) SAXS data. **o** Powder XRD patterns of ICO and Cu:ICO NCs. The pattern of vertical lines shown at the bottom corresponds to the powder XRD pattern of bulk CdO phase (JCPDS Card no. 03-065-2908). The error bars in (**k**) and (**m**) represent the standard deviation between measurements on the same sample. A minimum of three SEX-EDX measurements were performed over different spots to determine the average atomic ratios presented in (**k**). The NC size values shown in (**m**) were determined by statistical analysis of more than 1000 NCs from TEM images

ICO NCs were synthesized via thermal decomposition of cadmium acetylacetonate (Cd(acac)_2_) and indium acetate (In(ac)_3_) at elevated temperatures (~316 °C)^16^. 12 nm ICO NCs synthesized with 10% (atomic percent or at.%) indium input were used as the starting NCs for the majority of cation-exchange reactions (Fig. 1b). Typically, 0.2 mL of an ethanolic solution of CuCl_2_ was added to 1 mL stirred solution of ICO NCs in toluene (10 mg mL^−1^). The mixture was maintained at 60 °C for an hour after which NCs were isolated by centrifugation. Transmission electron microscopy (TEM) imaging revealed that NCs retained their initial size and shape uniformity after reacting with CuCl_2_ over the concentration range of 1–200 mM (Fig. 1c–e, 1m and Supplementary Figs. 1 and 2). This observation is further supported by the small-angle X-ray scattering (SAXS) data for different Cu-exchanged ICO (Cu:ICO) NCs (Fig. 1n). The distinctive ringing pattern and the excellent agreement between experimental and simulated SAXS results indicate that NCs are monodisperse at the ensemble level as they appear in TEM micrographs^46^. High-angle annular dark field (HAADF) scanning TEM (STEM) imaging proved the single-crystalline nature of the Cu:ICO NCs (Fig. 1f). Clear lattice fringes with measured interplanar spacing of about 0.27 nm can be indexed as (111) planes of the rocksalt CdO phase (Fig. 1f). Powder X-ray diffraction (XRD) and selected-area electron diffraction (SAED) patterns confirmed that the crystal structure of ICO NCs was retained after Cu doping at levels as high as 33% (Fig. 1o, Supplementary Fig. 1, Supplementary Table 1). Elemental mapping using electron energy-loss spectroscopy (EELS) in STEM mode revealed that Cu atoms were incorporated into NC lattice, showing insignificant Cu segregation at the NC surface (Fig. 1g–j). The elemental composition of different NCs was further quantified by using energy dispersive X-ray spectroscopy (EDX) performed on NC ensembles. As shown in Fig. 1k, higher Cu contents were detected when greater amounts of CuCl_2_ were used during reactions, which is accompanied by lower Cd concentrations and almost unchanged indium contents in NCs (Supplementary Table 1).

An interesting question that arises is whether Cu ions replaced indiscriminately the host and the dopant cations (i.e., Cd^2+^ and In^3+^ for ICO NCs), or substituted preferentially the host cations. To quantify the ion-selectivity during cation-exchange on pre-doped ICO NCs, the atomic ratios between Cd and In measured from Cu:ICO NCs vs. the concentration ratios between CuCl_2_ and Cd^2+^ in the starting ICO NCs (Cu/Cd %) were plotted. It is found that the Cd/In ratio decreases monotonically with increasing amounts of Cu (Fig. 1l). These results imply that Cu ions have a strong preference for replacing Cd^2+^ over In^3+^ of ICO NCs, as schematically illustrated in Fig. 1a.

### Optical properties and electronic structure of Cu:ICO NCs

**Fig. 2:**
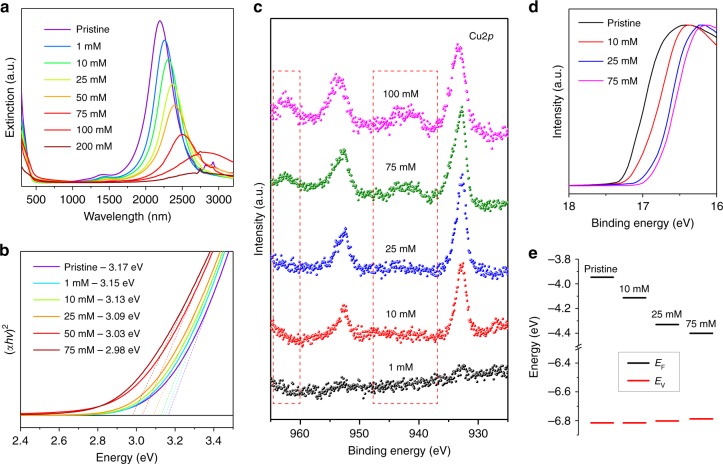
LSPR characteristics and electronic structure of Cu:ICO NCs. **a** UV–Vis–NIR spectra of ICO and Cu:ICO NCs. **b** Plots of (*αhν*)^2^ vs. photon energy for samples shown in (**a**). *α*: absorption coefficient. The dotted lines represent extrapolation from the linear region of the curves with the *x*-axis intercepts indicated in the legends. Plots for NCs reacted with 100 and 200 mM CuCl_2_ are not included because of the strong scattering background due to sample agglomeration. **c** High-resolution XPS spectra in the Cu2*p* region for Cu:ICO NCs. The dotted rectangles highlight the shake-up features attributed to Cu^2+^, whereas the two peaks centered at 933 and 952 eV, respectively, are signals arising from either Cu^+^ or Cu^2+^. **d** UPS spectra in the secondary electron cut-off region and **e** Fermi level *E*_F_ and valence band edge *E*_v_ (with respect to vacuum) for ICO and Cu:ICO NCs

We studied the effect of Cu incorporation on the optical properties of ICO NCs. Pristine ICO NCs (8.6% In) displayed a pronounced LSPR band centered at 2196 nm because of n-type In^3+^ dopants (Fig. 2a). UV–Vis–NIR spectra showed that as the concentration of CuCl_2_ increased, the LSPR band of Cu:ICO NCs shifted monotonically to longer wavelengths indicative of gradually decreased free carrier concentration (Fig. 2a, Table 1). A monotonic decrease in the optical direct bandgap energy was also observed at increasing levels of Cu incorporation, providing further evidence for a reduced occupation of low-lying energy levels within the conduction band at reduced carrier concentrations (Fig. 2b).

**Table 1:** Summary of LSPR characteristics and elemental analysis results for ICO and Cu:ICO NCs

[CuCl_2_] (mM)	Measured Cu (%)	*λ* (nm)	*E* (eV)	Δ*E* (eV)	*Q*	*ω*_p_ (cm^−1^)	*Γ* (cm^−1^)	*N*_e_ (10^20^ cm^−3^)
0	0	2196	0.565	0.105	5.38	14,423	753.13	6.29
1	0.3	2259	0.549	0.090	6.10	14,030	629.30	5.95
10	1.8	2309	0.537	0.090	5.97	13,726	632.40	5.69
25	4.2	2368	0.524	0.089	5.89	13,391	633.76	5.41
50	8.2	2399	0.517	0.102	5.07	13,183	739.52	5.25
75	10.9	2501	0.496	0.107	4.64	12,639	761.18	4.83
100	14.6	2822	0.440	0.165	2.67	11,231	1172.8	3.80

*Notes*: Cu concentrations (Cu/(Cu + Cd + In) %) were measured by using SEM-EDS. *λ*: LSPR peak wavelength, *E*: LSPR peak energy, Δ*E*: LSPR full width at half maximum, *Q*: LSPR quality factor (*E*/Δ*E*), *ω*_p_: bulk plasma frequency, *Γ:* free carrier damping constant, and *N*_e_: free carrier concentration

Because of the isovalency between Cd^2+^ and Cu^2+^ ions, substitution of Cd^2+^ by Cu^2+^ would result in minimal changes to the electron density of ICO NCs. To elucidate the electronic origin of LSPR redshifts, X-ray photoelectron spectroscopy (XPS) was employed to determine the oxidation state of Cu species in Cu:ICO NCs. As shown in Fig. 2c, two main peaks centered at ~932.8 eV (2*p*_3/2_) and ~952.7 eV (2*p*_1/2_), respectively, were routinely observed in the high-resolution Cu2*p* XPS spectra^47,48^. For reactions at low CuCl_2_ concentrations (≤25 mM), the lack of shake-up peaks associated with Cu^2+^ species in the XPS spectra suggested that only Cu^+^ ions were present in Cu:ICO NCs^47,48^. The shake-up features appeared for Cu:ICO NCs synthesized under high CuCl_2_ concentrations (≥75 mM), and deconvolution of the main Cu2*p*_3/2_ peak revealed the coexistence of Cu^+^ and Cu^2+^ ions (Supplementary Fig. 3, Supplementary Table 2). Meanwhile, XPS spectra of Cd3*d* and In3*d* regions remained essentially unchanged, suggesting that reactions between ICO NCs and CuCl_2_ did not alter the oxidation state of either Cd^2+^ or In^3+^ (Supplementary Fig. 4). Electron paramagnetic resonance (EPR) spectroscopy was also used to determine the oxidation state of Cu species within Cu:ICO NCs, given that Cu^2+^ is paramagnetic ([Ar]3*d*^*9*^) and EPR-active while Cu^+^ is diamagnetic ([Ar]3*d*^10^) and EPR-inactive^24,49^. Upon reaction with ICO NCs, the characteristic EPR signal from Cu^2+^ disappeared almost entirely (Supplementary Fig. 5). Collectively, these XPS and EPR results indicate that at low CuCl_2_ concentrations, Cu^2+^ ions were completely reduced to Cu^+^ ions resulting in compensated doping. At high CuCl_2_ concentrations, excess Cu^2+^ ions are likely to adsorb onto the surface of Cu:ICO NCs giving rise to the residual Cu^2+^ signal, although one cannot rule out the possibility of Cu^2+^/Cd^2+^ exchange on NC surface.

To establish quantitative correlations between composition and LSPR characteristics for different Cu:ICO NCs, we performed peak fitting of absorption spectra according to the Drude model (Table 1, Supplementary Fig. 6)^9,41^. The free carrier concentration *N*_e_ was found to decrease from 6.29 × 10^20^ cm^−3^ for ICO NCs (*λ*_initial_ = 2196 nm) to as low as 3.80 × 10^20^ cm^−3^ for Cu:ICO NCs. The LSPR quality factor (*Q*-factor), defined as the ratio of the peak energy to the full width at half maximum of the LSPR band, increased slightly at low Cu contents and decreased rapidly at high concentrations of Cu incorporation (Table 1). These results are consistent with the trend observed for the retrieved free carrier damping constant *Γ*, which shows reduced optical loss at low Cu levels (Table 1). Furthermore, ultraviolet photoelectron spectroscopy (UPS) measurements were performed to determine how Cu doping modifies the electronic structure and shifts the Fermi level of ICO NCs. As shown in Fig. 2d, the secondary electron cut-off shifted gradually to lower binding energies at higher Cu doping levels. As a result, the Fermi level *E*_F_ has moved from about −3.9 eV with respect to vacuum for initial ICO NCs to about −4.4 eV for Cu:ICO NCs after reacting with 75 mM CuCl_2_ (Fig. 2e). Meanwhile, the Fermi energy shifted closer to the valence band with higher Cu contents (Supplementary Fig. 7), which is consistent with the interpretation of increasingly compensated n-type ICO NCs upon addition of p-type Cu^+^ dopants. The Fermi energy values deduced from UPS measurements can be converted into electrochemical potential values relative to the standard hydrogen electrode (SHE), assuming an energy level value of −4.44 eV vs. vacuum for the SHE^50^. This conversion allows us to compare the redox properties of ICO NCs with various metallic cations. As shown in Supplementary Fig. 8, ICO NCs (8.6% In) with a Fermi level of −3.94 eV have an effective redox potential of −0.50 eV, which is more negative than the standard reduction potential of many cations including the Cu^2+^ ions. Therefore, it is thermodynamically feasible to reduce Cu^2+^ to Cu^+^ with electrons that are close to the Fermi level of ICO NCs. Similar redox reactions between Cu^2+^ and semiconductor NCs have been reported for CdS and CdSe^49,51^. However, ICO NCs exhibit a more negative reduction potential than many metal chalcogenide NCs and therefore are expected to be more reducing.

Oxidation of ICO NCs also occurred after their reactions with cations such as Au^3+^, Au^+^, and Hg^2+^, all of which possess a higher standard reduction potential and are therefore more oxidizing than Cu^2+^. Sizable redshifts in the LSPR frequency were consistently observed (Supplementary Fig. 9). However, SEM–EDX analysis revealed that negligible amounts of these cations became incorporated into the final ICO NCs (Supplementary Figs. 10–12, Supplementary Table 3). Instead, Au NCs were formed when either HAuCl_4_ or AuCl was used (Supplementary Figs. 10 and 11). Formation of white precipitates (presumably Hg_2_Cl_2_) were noticed after reacting ICO NCs with HgCl_2_. Therefore, the LSPR redshifts observed here were primarily caused by the electron transfer from ICO NCs to cations (i.e., Au^3+^, Au^+^, and Hg^2+^) in solution.

Spectral tuning of LSPR via selective cation-exchange reactions remained effective for ICO NCs doped with different concentrations of In^3+^ (Supplementary Fig. 13). When starting from 16.2% In-doped ICO NCs with an absorption band centered at 0.643 eV, the LSPR energy was lowered by nearly 200 meV upon reaction with CuCl_2_, which corresponds to nearly 50% decrease in electron density per NC (Supplementary Table 4).

It was observed that NCs had a strong tendency to agglomerate after reacting with a high concentration of CuCl_2_ (≥75 mM). This is likely due to the loss of Cd(oleate)_2_ ligands on NC surface. Displacement of the lead oleate ligands by AgNO_3_ have also been reported for the cation-exchange reaction between PbSe NCs and AgNO_3,_ causing PbSe NCs to lose colloidal stability when exposed to high concentrations of AgNO_3_^4^. To overcome this challenge, we tested another cation-exchange protocol that uses a mixture of didodecyldimethylammonium bromide (DDAB), dodecylamine (DDA), and CuCl_2_ as the Cu-precursor^3^. The reactions were carried out in a single organic phase (e.g., toluene), and Cu:ICO NCs exhibited excellent colloidal stability even after reaction with high concentrations of CuCl_2_. Reduction of Cu^2+^ to Cu^+^ and redshifts in LSPR were also realized (Supplementary Fig. 14). One drawback of using the DDAB–DDA–CuCl_2_ precursor is NCs appeared to become partially etched following reaction at high CuCl_2_ concentrations, limiting the range of Cu-doping level that can be studied (Supplementary Fig. 15).

### Kinetic study of cation-exchange reactions between ICO NCs and CuCl_2_

Two distinctive features were noticed for cation-exchange reactions between ICO NCs and CuCl_2_. First, the Cu^+^/Cd^2+^ exchange in ICO NCs took place much more slowly (tens of minutes) compared to similar exchange reactions taking place on metal chalcogenide NCs such as CdSe (typically within seconds)^42–44^. Second, there existed a strong preference for Cu^+^ to replace Cd^2+^ ions. Since both the overall concentration and the spatial distribution of dopants play an important role in controlling the LSPR characteristics of metal-oxide NCs, a thorough characterization of reaction intermediates will provide further mechanistic insight into the kinetics of dopant incorporation during cation-exchange reactions.

**Fig. 3:**
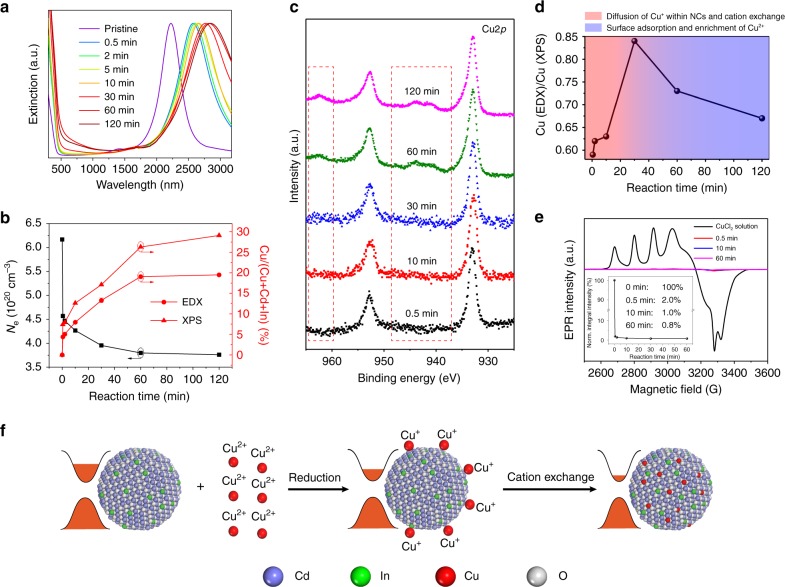
Kinetic study of cation-exchange reactions between ICO NCs (*λ*_initial_ = 2222 nm) and CuCl_2_. **a** UV–Vis–NIR spectra of Cu:ICO NCs isolated at different times from the reaction between ICO NCs and 100 mM CuCl_2_. **b** Free carrier concentrations retrieved from Drude fits to absorption spectra and Cu contents measured by SEM-EDS and XPS for Cu:ICO NCs isolated at different times from the reaction between ICO NCs and 100 mM CuCl_2_. **c** High-resolution Cu2*p* XPS spectra of Cu:ICO NCs isolated from the reaction between ICO NCs and 100 mM CuCl_2_ at different times. **d** Plot of the ratio between Cu concentrations determined by SEM-EDS and XPS measurements vs. reaction time. **e** Low-temperature (77 K) EPR spectra of 100 mM CuCl_2_ solution and ICO NCs after reacting with 100 mM CuCl_2_ for 0.5, 10, and 60 min. Inset: Time-dependent normalized integral peak intensities showing that nearly all Cu^2+^ ions were reduced to Cu^+^ within the first 30 s. **f** Proposed mechanistic scheme for the reaction between ICO NCs and CuCl_2_

Figure 3a presents the UV–Vis–NIR spectra of a series of aliquots extracted from the reaction mixture containing 100 mM CuCl_2_ (dissolved in ethanol) and ICO NCs (*λ*_initial_ = 2222 nm). A redshift in LSPR by about 76 meV was attained within 30 s of reaction. The LSPR continued to redshift by another 47 meV over the next 2 h (Table 2). The change in free carrier concentration retrieved from fitting of absorption spectra followed a similar trend, showing an initial sharp decline followed by a steady decrease at later stages of the reaction (Fig. 3b). Moreover, the shake-up peaks attributable to Cu^2+^ were not seen in the XPS spectra of NCs isolated within 30 min of reaction (Fig. 3c). These XPS data suggested that a rapid reduction (less than 30 s) of Cu^2+^ to Cu^+^ had occurred, and resulting Cu^+^ ions were subsequently incorporated into ICO NCs. A main peak at the kinetic energy of 915.7 eV was observed on the X-ray-induced Auger spectrum of Cu *LMM* region, which confirms the presence of Cu^+^, as well as the absence of Cu^0^ (Supplementary Fig. 16)^47,48^. The shake-up features of Cu^2+^ started to appear in the XPS spectra of NCs reacted for more than 1 h (Fig. 3c, Supplementary Fig. 17 and Supplementary Table 5).

**Table 2:** Summary of LSPR characteristics and elemental composition retrieved from kinetics study

Time (min)	Cu (%) measured by EDX	Cu (%) measured by XPS	Cu content (EDX)/Cu content (XPS)	*λ* (nm)	*E* (eV)	Δ*E* (eV)	*Q*	*ω*_p_ (cm^−1^)	*Γ* (cm^−1^)	*N*_e_ (10^20^ cm^−3^)
0	0	0	–	2222	0.558	0.094	5.94	14,290	676.47	6.17
0.5	4.4	7.4	0.59	2574	0.482	0.122	3.95	12,294	861.94	4.57
2	5.0	8.1	0.62	2596	0.478	0.125	3.82	12,167	865.16	4.47
10	8.0	12.6	0.63	2660	0.466	0.132	3.53	11,966	887.85	4.27
30	13.3	15.8	0.84	2764	0.449	0.145	3.10	11,879	1002.22	3.95
60	19.1	26.3	0.73	2820	0.440	0.165	2.67	11,443	1109.19	3.80
120	19.5	29.1	0.67	2848	0.435	0.158	2.75	11,210	1038.84	3.76

The elemental composition of NC intermediates was determined by using SEM–EDX and XPS analyses (Table 2, Supplementary Fig. 18). The Cu content in NCs increased gradually over time with a concomitant decrease in Cd content and a nearly unchanged indium content (Supplementary Fig. 18). XPS is a surface-sensitive technique because of the shallow escape depth of photoelectrons, whereas the sampling depth of SEM–EDX measurement is typically above 1 µm. The Cu concentrations measured by XPS for different NC intermediates were found to be consistently higher than the values obtained from SEM–EDX analysis, indicative of the presence of a Cu-rich near-surface region within Cu:ICO NCs (Fig. 3b). The extent of Cu’s surface enrichment can be estimated by taking the ratio between atomic percent values obtained from XPS and EDX measurements. As depicted in Fig. 3d, the Cu(EDX)/Cu(XPS) ratio increased monotonically during the first 30 min of reaction. We speculate that this period corresponds to continued migration of Cu^+^ ions from the surface of ICO NCs to their cores as the Cu^+^/Cd^2+^ exchange reaction progresses. The Cu(EDX)/Cu(XPS) ratio started to decrease after 30 min of reaction, which was attributed to the adsorption of Cu^2+^ ions onto NC surface when taking into account the XPS results shown in Fig. 3c. Low-temperature (77 K) EPR experiments were also performed to monitor changes in the oxidation state of copper. EPR tubes containing reaction aliquots were plunged into liquid N_2_ enabling rapid freezing of extracted solutions. More importantly, this step allowed the cation-exchange reaction between ICO NCs and Cu^+^ ions to be successfully halted (Supplementary Fig. 19). The integral intensity of Cu^2+^ EPR signal dropped by 98% within the initial 30 s when 100 mM CuCl_2_ was used, demonstrating that almost all Cu^2+^ were reduced on the time scale of several tens of seconds (Fig. 3e).

Taken together, a mechanistic picture has emerged for the reaction between ICO NCs and CuCl_2_. The reaction can be divided into two distinct stages, as schematically illustrated in Fig. 3f. The first stage is the rapid reduction of Cu^2+^ by electron-rich ICO NCs driven primarily by the difference between their reduction potentials. This redox reaction lowered the electron density of ICO NCs and produced a redshift in LSPRs. The loss of electrons can induce a net positive charge on the NC surface, which are charge balanced via adsorption of Cl^−^ ions. This argument is supported by the XPS results of thoroughly cleaned NC intermediates extracted at various time intervals (Supplementary Fig. 20). A rapid rise in chlorine content was detected for NCs retrieved after 30 s of reaction. The second reaction stage is characterized by the Cu^+^/Cd^2+^ exchange process, which continues to reduce the free carrier concentration and shift the LSPR to longer wavelengths (Fig. 3f).

It is important to point out that the Cu^+^/Cd^2+^ exchange can also be realized by running the reaction with a Cu^+^ precursor, such as tetrakis(acetonitrile) copper(I) hexafluorophosphate ([Cu(CH_3_CN)_4_]PF_6_) or DDAB–DDA–CuCl (Supplementary Figs. 21 and 22). Elemental analysis revealed that Cu^+^ ions replaced preferentially the Cd^2+^ ions of ICO NCs, leaving the concentration of In^3+^ ions essentially unvaried (Supplementary Fig. 21b). Starting from ICO NCs with *λ*_initial_ = 2196 nm, the magnitude of LSPR redshift near the end of reaction was smaller (52 meV with [Cu(CH_3_CN)_4_]PF_6_ and 32 meV with DDAB–DDA–CuCl) than that attainable with CuCl_2_ (123 meV). These results are consistent with the fact that the step of Cu^2+^ reduction by ICO NCs is missing when using a Cu^+^ precursor.

### Cation-exchange reactions in other metal-oxide/cation combinations

**Fig. 4:**
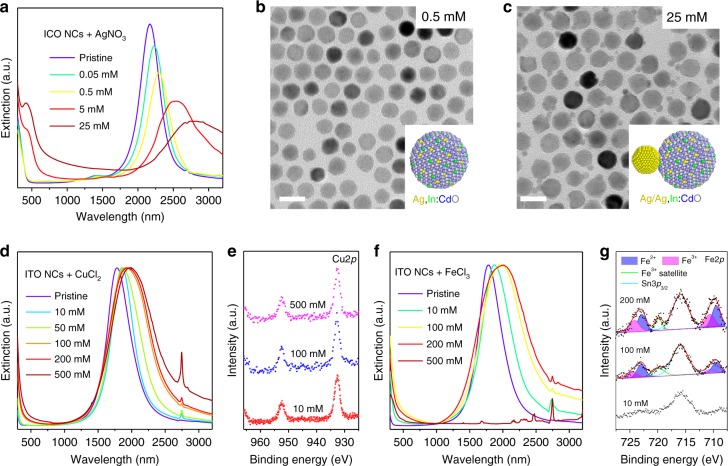
Versatility of cation-exchange reactions for LSPR spectral tuning in doped metal-oxide NCs. **a** UV–Vis–NIR spectra. **b**, **c** TEM images of Ag:ICO NCs synthesized by reacting ICO NCs with various concentrations of AgNO_3_ for 1 h. Scale bars: 20 nm. **d** UV–Vis–NIR spectra and **e** XPS spectra of Cu:ITO NCs synthesized by reacting ITO NCs with various concentrations of CuCl_2_. **f** UV–Vis–NIR spectra and **g** XPS spectra of Fe:ITO NCs synthesized by reacting ITO NCs with various concentrations of FeCl_3_

Having established the feasibility of using cation-exchange reaction to tune LSPR in ICO NCs, we further evaluated the generality of this approach with other combinations of cations and metal-oxide NCs. Similar to Cu^+^, Ag^+^ is also a p-type impurity when substitutionally doped into various metal oxides. Starting from ICO NCs with *λ*_initial_ = 2170 nm, the LSPR peak became more redshifted upon reacting with increasing concentrations of AgNO_3_ (Fig. 4a). At low AgNO_3_ concentrations, no obvious changes in NC’s size and shape were observed based on TEM imaging (Fig. 4b). STEM-EELS elemental mapping suggested that a considerable amount of Ag was incorporated into NCs (Supplementary Fig. 23a–e). Furthermore, SEM–EDX analysis indicated that Ag^+^ ions had replaced preferentially the Cd^2+^ ions of ICO NCs (Supplementary Table 6). At high AgNO_3_ concentrations, reactions between ICO NCs and AgNO_3_ yielded predominantly heterodimer NCs composed of separate Ag and ICO crystalline domains (Fig. 4c, Supplementary Fig. 23f–j), and yet the concentration of Ag within the ICO domain appeared to be much lower than that of Ag-doped ICO NCs shown in Fig. 4b. The formation of Ag NCs was also evidenced by the appearance of the second LSPR band centered at ~440 nm (Fig. 4a). It is plausible that at high AgNO_3_ concentrations, the fast reduction kinetics of Ag^+^ to Ag^0^ promotes the nucleation and growth of Ag NCs. It is also conceivable, given their high mobility in solids, that Ag^+^ ions are first incorporated into ICO NC lattice through the Ag^+^/Cd^2+^ exchange, and later migrate out to the surface and grow into Ag NCs^52^. Work is currently underway to gain a deeper mechanistic understanding of how the relative kinetics of cation-exchange and redox reactions dictates the reaction pathways between ICO NCs and Ag^+^ ions.

We further expanded this study to other types of extrinsically doped metal-oxide NCs. ITO NCs represent another widely studied example of degenerately doped metal-oxide NCs^13,15,20,41,53^. Reactions between ITO NCs and CuCl_2_ were performed in a manner analogous to the Cu-exchange reactions on ICO NCs. Typically, 0.2 mL ethanol solution of CuCl_2_ were added to 1 mL toluene solution of ITO NCs (10 mg mL^−1^), and the reaction was allowed to proceed at 60 °C for 1 h. As shown in Fig. 4d, the LSPR of 6.5% Sn-doped ITO NCs, initially peaked at 1784 nm, became increasingly red-shifted after reacting with higher accounts of CuCl_2_. TEM and powder XRD showed that the size, shape, and crystal phase of ITO NCs were well preserved (Supplementary Fig. 24a–d). The lack of shake-up features in the Cu2*p* XPS spectra further indicated that the reduction of Cu^2+^ by ITO NCs had taken place and Cu^+^ species were present within Cu:ITO NCs (Fig. 4e). Meanwhile, the oxidation states of both In and Sn remained unaltered (Supplementary Fig. 24e, f). Notably, SEM–EDX analysis of ITO and Cu:ITO NCs suggested a preferential exchange between Cu^+^ ions and the In^3+^ host cations (Supplementary Table 7).

Fe-doped ITO NCs (Fe:ITO) were also synthesized via selective Fe^2+^/In^3+^ exchange upon reacting ITO NCs (6.5% Sn) with FeCl_3_. Both XPS (Fig. 4g) and SEM–EDX measurements (Supplementary Table 8) showed that higher Fe contents were measured in Fe:ITO NCs at higher FeCl_3_ concentrations, accompanied by lowering in LSPR energy (Fig. 4f). Signals corresponding to both Fe^2+^ and Fe^3+^ species were detected in the XPS spectra of Fe:ITO NCs, suggesting that Fe^3+^ ions were partially reduced by ITO NCs to Fe^2+^ ions^54^. However, complications such as spectral overlap between the Sn3*p*_3/2_ and the Fe2*p* region (Fig. 4g) and low signal-to-noise ratio of the Fe3*p* spectra (Supplementary Fig. 25h) had prevented a reliable quantification of the relative population between Fe^2+^ and Fe^3+^ species.

Cation-exchange reactions between host cations and Cu^+^ ions were also feasible when starting from unintentionally doped metal-oxide NCs, such as ZnO and In_2_O_3_ (Supplementary Fig. 26). XPS data suggested that the initial Cu^2+^ were mostly reduced to Cu^+^ by metal-oxide NCs (Supplementary Fig. 27). The electrons participating in the redox process likely stem from native defects, especially oxygen vacancies that have been shown to induce n-type behavior in unintentionally doped ZnO^55,56^ and In_2_O_3_^57^. The monotonic decrease in the optical direct bandgap for ZnO and In_2_O_3_ NCs after reacting with increasing amounts of CuCl_2_ is consistent with the interpretation that adding p-type Cu^+^ dopants into NC lattice can lower the Fermi level and enhance NC absorption at longer wavelengths (Supplementary Fig. 27a, b).

### Tuning LSPR energies of metal-oxide NCs via sequential cation-exchange reactions

**Fig. 5:**
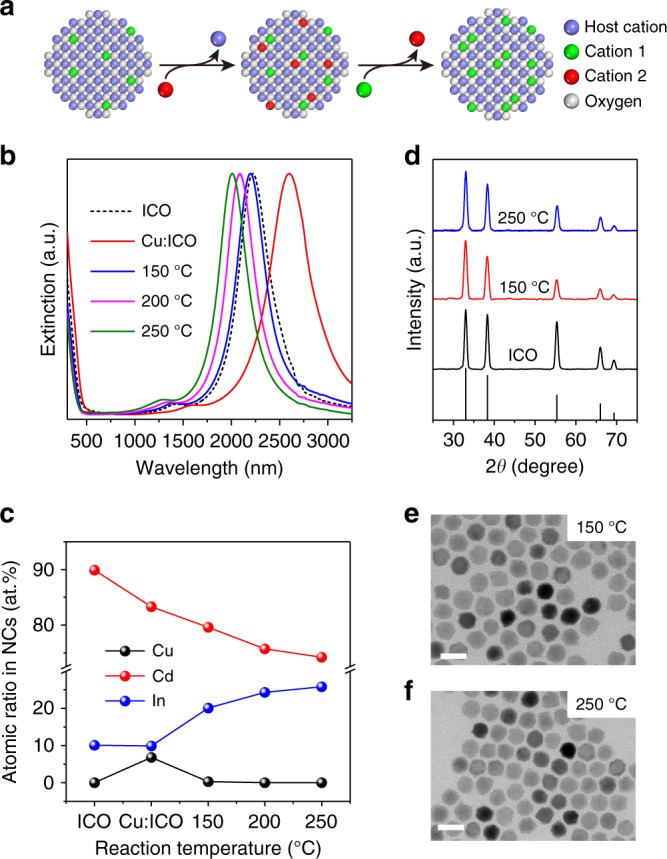
Tuning LSPR energies of ICO NCs via sequential cation-exchange reactions. **a** Proposed scheme for the sequential cation-exchange reactions. **b** UV–Vis–NIR spectra and **c** elemental composition determined by SEM-EDS analysis, and **d** powder XRD patterns for ICO NCs, Cu:ICO NCs and NCs obtained by reacting Cu:ICO NCs with In(ac)_3_ at different temperatures. **e**, **f** TEM images of NCs synthesized by reacting Cu:ICO NCs with In(ac)_3_ at (**e**) 150 °C and (**f**) 250 °C. Scale bars: 20 nm

Selective Cu^+^/Cd^2+^ exchange with ICO NCs allows for the introduction of p-type Cu^+^ dopants into the CdO lattice while leaving the original n-type In^3+^ dopants largely intact. We hypothesize that if incorporated Cu^+^ ions can be selectively replaced by In^3+^ ions in a subsequent reaction step, the net result would be more In^3+^ are added to NCs, which can lead to enhanced n-type character and thereby shift the LSPR to even higher frequencies than the starting ICO NCs (Fig. 5a). In fact, such In^3+^/Cu^+^ exchange has recently been demonstrated in the synthesis of CuInX_2_ (X = S, Se) NCs from Cu_2_X NCs^58,59^, and the synthesis of phase-pure InP NCs from Cu_3−*x*_P NCs^60^.

To test our hypothesis, ICO NCs with *λ*_initial_ = 2222 nm were first subjected to the Cu^+^/Cd^2+^ exchange reaction, generating Cu:ICO NCs with a redshifted LSPR centered at 2598 nm (Fig. 5b). These Cu:ICO NCs were then allowed to react with In(ac)_3_ dissolved in trioctylphosphine oxide (TOPO) at a certain temperature for 10 min. The atomic percentage of copper for NCs after reaction at 150 °C reached nearly zero according to SEM–EDX analysis (Fig. 5c, Supplementary Table 9), suggesting that the In^3+^/Cu^+^ exchange had taken place. The LSPR peak position blue-shifted from 2598 nm (for Cu:ICO NCs) to 2196 nm (Fig. 5b), which is consistent with the expected increase in carrier concentration as a result of indium incorporation. SEM–EDX analysis revealed that In^3+^ ions had also replaced some Cd^2+^ ions during the reaction between In(ac)_3_ and Cu:ICO NCs (Fig. 5c, Supplementary Table 9). Larger LSPR blue-shifts were attained when the reaction was carried out at higher temperatures (Fig. 5b). The magnitude of the LSPR blue-shift is well correlated with the measured indium content that is largely determined by the extent of In^3+/^Cd^2+^ exchange at different temperatures (Fig. 5c, Supplementary Table 9). The use of TOPO as the solvent was found to play a key role in preserving the structural integrity of ICO NCs and in suppressing the formation of the In_2_O_3_ phase (Fig. 5d–f). When 1-octadecene (ODE) was used as the solvent, ICO NCs appeared to be corroded with concomitant formation of irregularly shaped In_2_O_3_ NCs based on XRD analysis (Supplementary Fig. 28). It has been argued that TOPO can bind strongly to In^3+^ ions, which might increase the nucleation barrier for In_2_O_3_ NCs^61^. Furthermore, trioctylphosphine (TOP) was found to be necessary to facilitate the In^3+^/Cu^+^ and the In^3+^/Cd^2+^ exchanges via solvation of Cu^+^ and Cd^2+^ ions in solution while retaining structural integrity of individual NCs (Supplementary Fig. 29).

## Discussion

Our results clearly demonstrate that cations including Cu^+^, Ag^+^, among others, can be successfully incorporated into various pre-doped n-type metal-oxide NCs via preferential cation-exchange with NC’s host cations. The vast majority of newly added cations are p-type dopants and the process bears close resemblance to compensation doping in bulk semiconductors. Reactions between NCs and Cu^2+^ are characterized by two distinct stages: the initial rapid reduction of Cu^2+^ to Cu^+^ followed by the cation-exchange process between Cu^+^ and host cations. Both steps cause a reduction in electron density, producing a greater LSPR redshift than that attainable from reactions starting with a Cu^+^ precursor. The ion-selectivity during cation-exchange reactions can be rationalized by considering the bond dissociation energy (BDE)^43,62^. Taking ICO NCs as an example, the average BDE value of Cd–O bond (236 ± 84 kJ mol^−1^) has been determined to be considerably smaller than that of the In–O bond (346 ± 30 kJ mol^−1^). This difference in BDE suggests that thermodynamically it would be more difficult to break the In–O bond than the Cd–O bond, and the resulting lower energy barrier for removal of Cd^2+^ vs. In^3+^ is in good agreement with experimentally observed preference for the Cu^+^/Cd^2+^ exchange over the Cu^+^/In^3+^ with ICO NCs. A similar argument can be made for ion-selectivity with ITO NCs by considering the even higher BDE value for the Sn–O bond (528 kJ mol^−1^). Future work to validate this mechanism includes cation-exchange reactions on doped oxide NCs for which the BDE of dopant–oxygen bond is smaller than that of the host–cation–oxygen bond.

Colloidal synthesis of doped NCs often demands carefully balanced reaction conditions to facilitate the incorporation of dopants into host lattice. Although a wide range of compositions for doped plasmonic oxide NCs have become accessible via the most commonly used co-thermolysis method, the preparation of Cu-doped and Ag-doped plasmonic metal-oxide NCs has remained particularly difficult. Taking Cu:ICO NCs as an example, our attempts to directly synthesize these NCs through thermal decomposition of Cd(acac)_2_ and different Cu precursors had merely produced sub-µm-sized CdO NCs with no signatures of Cu-doping (Supplementary Fig. 30). In contrast, the cation-exchange method developed here offers a facile “conversion chemistry” route to create NCs with previously inaccessible compositions and topologies. The Ag–ICO heterodimer NCs with dual LSPR bands represent another example that further illustrates this point (Fig. 4a, c). Although previous work has shown that a variety of metal–ICO heterodimer NCs can be made by growing ICO NCs onto preformed seed NCs including Au, Pt, Pd, and FePt, Ag NCs do not survive the high temperature at which ICO NCs are formed (~316 °C) and hence only ICO NCs are produced in the presence of added Ag NCs^63^.

Cation-exchange reactions also enable sophisticated postsynthetic tuning of LSPR wavelength for metal-oxide NCs. We have found that varying either the concentration of dopant ions exposed to preformed metal-oxide NCs or reaction time allows the magnitude of LSPR shifts to be controlled with milli-electron volt precision. Since variations in LSPR peak energy are routinely found for metal-oxide NCs synthesized under nominally identical input of dopants, we argue that the combination of direct thermolysis of mixed precursors and cation-exchange reactions constitutes a powerful toolkit for the targeted synthesis of metal-oxide NCs with desired LSPR characteristics. The ability to readily tune LSPR frequency to match a vibrational mode of interest without compromising the LSPR *Q*-factor can be beneficial for boosting the sensitivity in SEIRAS.

In summary, we have developed an effective cation-exchange method for the incorporation of various cationic dopants into preformed metal-oxide NCs while preserving their size, shape, and crystal structure. We demonstrate that successful addition of p-type dopants, such as Cu^+^, Ag^+^, and Fe^2+^ into n-type metal-oxide NCs, which may not be feasible using alternative synthetic methods, leads to programmable LSPR redshifts due to dopant compensation. We further show that enhanced n-type doping can be realized via rationally designed sequential cation-exchange reactions mediated by the Cu^+^ ions, yielding metal-oxide NCs with the ensemble LSPR energy surpassing that of starting NCs. Cation-exchange transformations allow doped metal-oxide NCs synthesized by existing methods to be used as templates to create compositionally tunable NCs that exceed existing performance limits in LSPR wavelength range. The ability to postsynthetically alter the doping profile of individual NCs opens the door to a multitude of new opportunities to deepen understanding of the relationship between local structural details and plasmonic properties of metal-oxide NCs.

## Methods

### Chemicals

Cadmium(II) acetylacetonate (Cd(acac)_2_, ≥99.9%), zinc stearate (ZnSt_2_, technical grade), tetrakis(acetonitrile)copper(I) hexafluorophosphate ([Cu(CH_3_CN)_4_]PF_6_, 97%), copper(I) chloride (CuCl, 99%), silver nitrate (AgNO_3_, 99%), hydrogen tetrachloroaurate trihydrate (HAuCl_4_·3H_2_O, 99.9%), gold(I) chloride (AuCl, 99.9%), mercury(II) chloride (HgCl_2_, 99.5%), iron(III) chloride (FeCl_3_, 97%), oleic acid (OLAC, 90%), ODE (90%), 1-dodecanol (DDOL, 98%), TOP (97%), oleylamine (OLAM, 70%), tetrachloroethylene (TCE, 99%) were purchased from Sigma-Aldrich. Indium acetate (In(ac)_3_, 99.99%), copper(II) chloride, (CuCl_2_, anhydrous, >98%), was purchased from Alfa Aesar. DDAB (98%) and oleyl alcohol (>60%) were purchased from TCI America. DDA (98%) and TOPO (99%) were purchased from Acros Organics. Anhydrous solvents were obtained from a column solvent purification system.

### Synthesis of ICO NCs

ICO NCs were synthesized according to previously reported method under nitrogen atmosphere using standard Schlenk line techniques^16^. In a typical reaction, 3 mmol of metal precursors (e.g., for 10% ICO, 2.7 mmol of Cd(acac)_2_, and 0.3 mmol of In(ac)_3_), 15 mmol of OLAC and 100 mL of ODE were loaded into a 250 mL three-neck flask. After degassing under vacuum for 1 h at 100 °C, the mixture was heated rapidly to reflux (~316 °C). The color of the solution turned dark brown after about 30 min of refluxing, indicating the formation of ICO NCs. The reaction flask was air-cooled to room temperature 10 min after the initial color change, and NCs were isolated by precipitation with isopropanol and centrifugation at 6000 rpm for 5 min. The NCs were re-dispersed in 30 mL of hexane in the presence of 0.2 mL of OLAM and 0.2 mL of OLAC, and centrifuged at 3000 rpm for 3 min to remove large aggregates and Cd NC byproducts. Following another round of isopropanol precipitation and centrifugation, the ICO NCs were finally dispersed in anhydrous toluene (10 mg mL^−1^). It is worth mentioning that the incorporated In content or In/(In + Cd) atomic ratio for ICO NCs determined by elemental analysis does not precisely follow the input In content within mixed metal precursors. Taking 10% In input as an example, the In/(In + Cd) atomic ratio for ICO NCs from different batches was found to vary in the range from 8.5% to 11.0%.

### Synthesis of ITO and In_2_O_3_ NCs

ITO and In_2_O_3_ NCs were synthesized according to previously reported method^22^. In a typical reaction, 1.8 mmol of In(ac)_3_ and 0.2 mmol of Sn(ac)_4_ were mixed with 4 mL of OLAC in a 25 mL flask. After degassing under vacuum for 1 h at 100 °C, the mixture was heated at 150 °C for 30 min to promote the formation of metal oleate precursors. In another reaction flask, 26 mL of oleyl alcohol was vacuum dried at 100 °C for 1 h before being heated to 290 °C under N_2_ atmosphere. Afterwards, the metal precursor solution was added dropwise via a syringe pump (~ 0.35 mL min^−1^) into the flask containing oleyl alcohol. After completion of injection, the reaction mixture was kept at 290 °C for another 10 min before being cooled to room temperature. A similar procedure was employed for the synthesis of In_2_O_3_ NCs using 2 mmol of In(ac)_3_ as the metal precursor.

### Synthesis of ZnO NCs

ZnO NCs were synthesized according to previously reported method^38^. In a typical reaction,1.89 g of ZnSt_2_, 4.8 g of DDOL, 2.65 g of OLAC, and 21 mL of ODE were loaded into a 100 mL three-neck flask. After vacuum degassing at 60 °C for 10 min, the mixture was heated at 140 °C for 30 min to promote complete dissolution of ZnSt_2_. The solution temperature was subsequently raised to 250 °C at a heating rate of ∼4 °C min^−1^ and was kept at 250 °C for 3 h before being cooled down to room temperature.

### Cation-exchange reactions

Cation-exchange reactions of metal-oxide NCs were carried out inside a N_2_-filled glovebox using anhydrous solvents. Typically, 0.2 mL of ethanolic solutions of the appropriate metal salts, such as CuCl_2_ with different concentrations (from 1 to 500 mM) were prepared, to which 1 mL toluene solution of metal-oxide NCs (10 mg mL^−1^) was added. The mixture was then heated to 60 °C under stirring using a hotplate. The reaction was allowed to proceed for 1 h at 60 °C, and was halted by the injection of 4 mL of methanol. The NCs was isolated via centrifugation at 6000 rpm for 3 min and were dissolved in TCE or toluene for further characterization. One exception to this general procedure is methanol was used to dissolve [Cu(CH_3_CN)_4_]PF_6_ because of its poor solubility in ethanol.

For Cu-exchange reactions that involves DDAB–DDA, 2 mmol of CuCl_2_ or CuCl together with 1.2 g of DDAB and 0.8 g of DDA were dissolved in 10 mL of toluene at 80 °C under stirring. The cation-exchange process was performed by adding a certain amount of the DDAB–DDA–CuCl_2_ (or CuCl) solution into 1 mL of toluene solution of ICO NCs (20 mg mL^−1^). The reaction mixture was heated at 60 °C under stirring for 1 h after which NCs were isolated via centrifugation.

### Sequential cation-exchange reactions with ICO NCs

Cu:ICO NCs were first synthesized via a cation-exchange reaction between ICO NCs and the DDAB–DDA–CuCl_2_ precursor, as detailed in the last section. The Cu:ICO NCs were isolated by methanol precipitation followed by centrifugation and were re-dispersed in 2 mL of TOP. In a separate 25 mL three-neck flask, indium precursor was prepared by dissolving 0.2 mmol of In(ac)_3_, 0.5 mmol of OLAC (0.16 mL) in 5 g of TOPO at 120 °C. After degassing under vacuum for an hour, the solution mixture was heated to 150 °C under nitrogen and Cu:ICO NC solution was rapidly injected. The reaction was allowed to proceed for another 10 min at a given temperature (150, 200, 250, 300 °C). Afterwards, NCs were isolated by isopropanol precipitation and centrifugation at 6000 rpm for 5 min and were dissolved in TCE for further study.

### Drude fitting of LSPR absorbance

Drude fitting of LSPR peaks in the UV–Vis–NIR spectra was performed by using a custom written Matlab script. The absorbance (*A*) of NC solutions is determined by the Beer−Lambert law^53^:

1 $$A = \frac{{N\sigma _{\mathrm{A}}l}}{{{\mathrm{ln}}{\mathrm{(10)}}}}$$

where *N* is the NC concentration in solution, and *l* is the path length of the cuvette. According to Mie theory^25^, the absorption cross section (*σ*_A_) of spherical NCs can be calculated as:

2 $$\sigma _{\mathrm{A}} = 4{\mathrm{\pi }}{\mathbf{k}}r^3{\mathrm {Imag}}\left( {\frac{{\varepsilon (\omega ) - \varepsilon _{\mathrm{m}}}}{{\varepsilon (\omega ) + 2\varepsilon _{\mathrm{m}}}}} \right)$$

where **k** is the wave vector of the incident light and ${\mathbf{k}} = \sqrt {\varepsilon _{\mathrm{m}}} \omega /c$, *r* is the NC radius, *ε*(*ω*) is the dielectric function of NCs, and *ε*_m_ is the dielectric constant of the medium (for TCE, *ε*_m_ = 2.268). The frequency-dependent dielectric function is given by:

3 $$\varepsilon _{(\omega )} = \varepsilon _\infty - \frac{{{\omega _{\mathrm{p}}}^2}}{{(\omega ^2 + {\mathrm{i}}\omega \varGamma )}}$$

where *ε*_∞_ is the high-frequency dielectric constant (for ICO, *ε*_∞_ = 5.5)^34^, *ω*_p_ is the bulk plasma frequency, and *Γ* is the free carrier damping constant. By fitting the LSPR absorption spectrum with the Drude model, *ω*_p_ and *Γ* can be retrieved. The free carrier concentration *N*_e_ can then be calculated using the formula^9,41^:

4 $$\omega _{\mathrm{p}} = \sqrt {\frac{{N_{\mathrm{e}}e^2}}{{m^ \ast \varepsilon _0}}}$$

where *e* is the elementary charge, m* is the effective mass of electrons (for ICO, m* = 0.27*m*_0_)^64^, and *ε*_0_ is the vacuum permittivity.

### Structural and optical characterization

Low-magnification TEM images and SAED patterns were acquired on a JEOL JEM 1400 plus microscope operating at 120 kV with a LaB_6_ filament. TEM samples were prepared by drop-casting ~10 µL of NC solution onto a 300-mesh carbon-coated copper grid (Ted Pella). STEM-HAADF images (spatial resolution of ~1 Å) and STEM-EELS elemental maps were acquired on an aberration-corrected Hitachi HD 2700C STEM microscope equipped with a parallel EELS detector (Gatan Enfina-ER). Samples were prepared using holey 400-mesh Cu grids coated with ultrathin carbon films (Ted Pella). The STEM-EELS mapping was performed at a collection angle of 26.7 mrad with a dispersion of 1.25 eV per channel. The spectrum images are 72 by 72 pixels with a pixel size of 5.6 Å. EDX analysis was conducted with an X-Max 50 mm^2^ silicon drift detector (Oxford Instruments) interfaced either to a Carl Zeiss Auriga SEM or to a JEOL JEM 1400 plus TEM via the AZtec software package. For EDX analysis, NCs were thoroughly cleaned with the precipitation–dissolution procedure for 4–6 times before being deposited onto a silicon substrate (for SEM–EDX) or a TEM grid (for TEM–EDX).

Solution-phase UV–Vis–NIR absorption spectra were recorded on a Varian Cary 5000 spectrometer. Fourier-transform infrared spectroscopy (FTIR) spectra were recorded on a Bruker Vertex 70V FTIR spectrometer in transmission mode using a Harrick demountable liquid cell. Determination of the cadmium content for ICO NCs was carried out on an Agilent 7700 ICP-MS. Samples for ICP-MS measurements were prepared by digesting NCs with aqua regia followed by dilution with ultrapure water. Powder XRD patterns were collected on a PANalytical Empyrean diffractometer with a copper target. SAXS experiments were performed at beamline station 12-ID-C of Advanced Photon Source (Argonne National Laboratory, Argonne, IL) with the incident X-ray energy of 12 keV (*λ* = 1.0332 Å). SAXS data was collected via a 2D CCD detector at a sample-to-detector distance of 2 m with exposure times ranging from 0.01 to 1 s. Analysis and modeling of SAXS data were performed by using the Irena software package^65^. Models were fit using the size distribution model with the lognormal distribution. SAXS data for NCs reacted with high concentrations of CuCl_2_ (75 and 100 mM) showed a slight distortion in the low *q* range below 0.04 Å^−1^, indicating NC aggregation^46^. To compensate for the effects caused by particle aggregation, models were calculated using interferences as the structure factor^46^. The structure factor parameter of 156 (141) for 75 mM (100 mM) CuCl_2_-reacted samples was used to describe the relative positions between NCs.

### XPS and UPS

XPS analysis was performed on a PHI 5000 *VersaProbe*^TM^ II instrument equipped with a focused monochromatic Al K(alpha) source. The instrument base pressure was about 8 × 10^–10^ Torr. A beam size of about 200 μm and an X-ray power of 50 W at 15 keV was used for all experiments. The C 1*s* peak calibrated at 284.4 eV was used as an internal standard for the binding energy scale. The PHI’s dual beam charge neutralization system was used for all XPS measurements. UPS spectra were recorded with a He(I) and He(II) excitation source (21.2 and 40.8 eV, respectively) and a pass energy of  0.58 eV in the UHV chamber of the XPS instrument. NC samples were drop casted onto a piece of gold foil for UPS testing.

### EPR spectroscopy

EPR spectra were acquired on a Bruker EMX X-band EPR spectrometer with an ER 4119 HS cavity (9.840 GHz at room temperature) using 100 kHz field modulation (modulation amplitude: 6 G). A 150 mL Suprasil offset liquid nitrogen dewar flask (Wilmad-LabGlass) was used for low-temperature measurements. Individual EPR tubes were filled with ~0.2 mL of solution inside a glovebox and were placed at the same position of the resonant cavity for EPR spectral acquisition.

## Supplementary information

Supplementary Information

## Data Availability

All necessary data generated or analyzed during this study are included in this published article, and other auxiliary data are available from the corresponding authors upon request.

## Code availability

The Matlab scripts necessary to reproduce our results are available from the corresponding author (X.Y.) upon request.
