# Supplementary material for: Tuning infrared plasmon resonances in doped metal-oxide nanocrystals through cation-exchange reactions
Source: Nat Commun. 2019 Mar 27;10:1394. doi: 10.1038/s41467-019-09165-2 (PMC6437201; doi:10.1038/s41467-019-09165-2)
Supplement: Supplementary file 1 — Supplementary Information [file 41467_2019_9165_MOESM1_ESM.pdf]

Supplementary Information

# Tuning infrared plasmon resonances in doped metal-oxide nanocrystals through cation-exchange reactions

Liu et al.

# Supplementary Figures

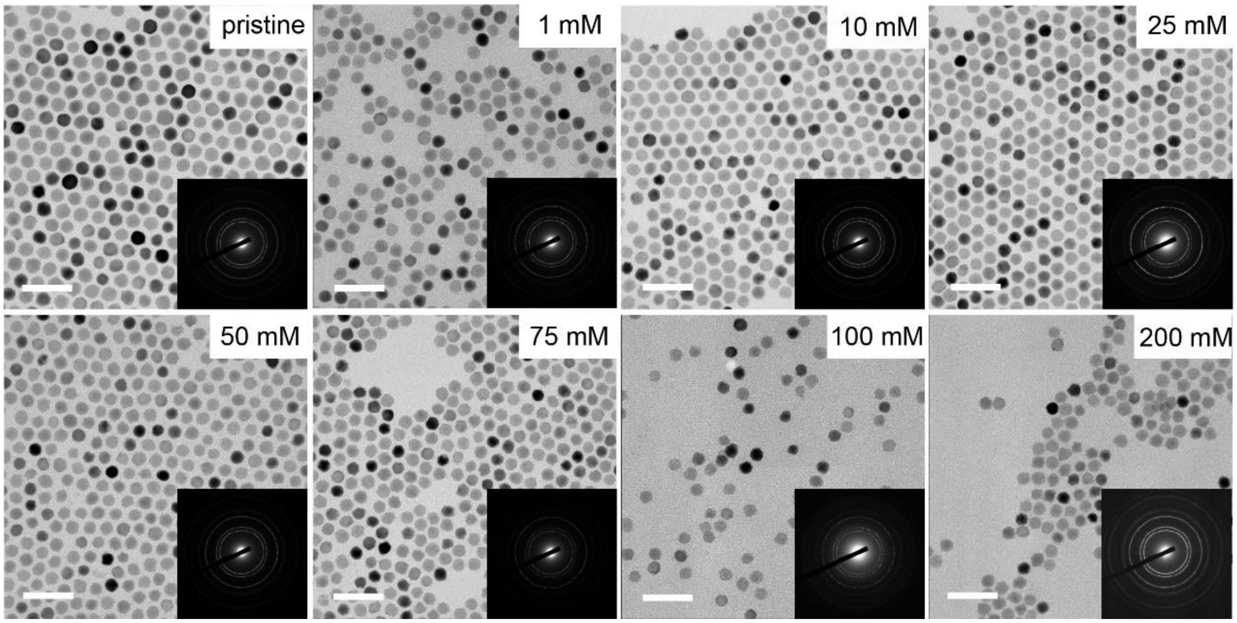


**Supplementary Figure 1 | TEM characterization.** Additional TEM images of ICO ($\lambda_{initial}$ = 2196 nm) and Cu:ICO NCs. Insets show the corresponding SAED patterns. Scale bars: 50 nm.

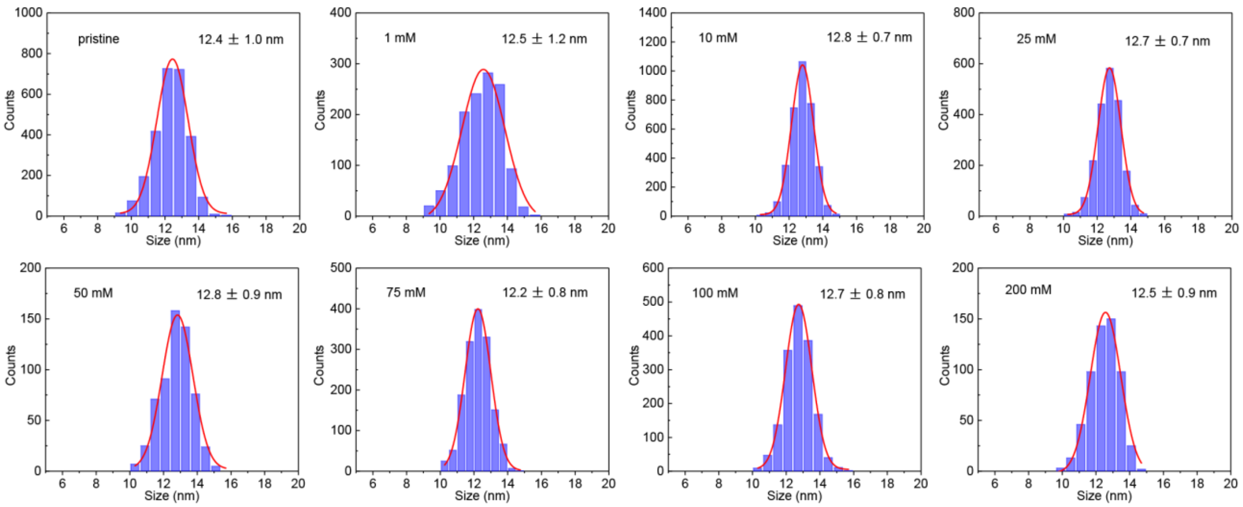


**Supplementary Figure 2 | Statistical analysis of NC sizes before and after cation-exchange reactions with $CuCl_2$.** Size distribution histograms obtained from image analysis of TEM micrographs shown in Supplementary Figure 1. The red curves represent Gaussian fit to the distribution histogram.

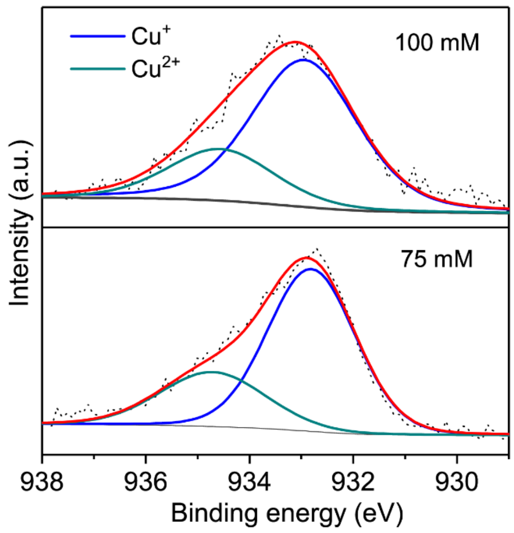


**Supplementary Figure 3 | Determination of the atomic ratio between $Cu^+$ and $Cu^{2+}$ from XPS spectra.** Deconvolution of the XPS data presented in Figure 2c for Cu:ICO NCs synthesized by reacting ICO NCs ($\lambda_{initial}$ = 2196 nm) and high concentrations of $CuCl_2$. Peaks corresponding to $Cu^+$ and $Cu^{2+}$ species are well resolved after deconvolution.

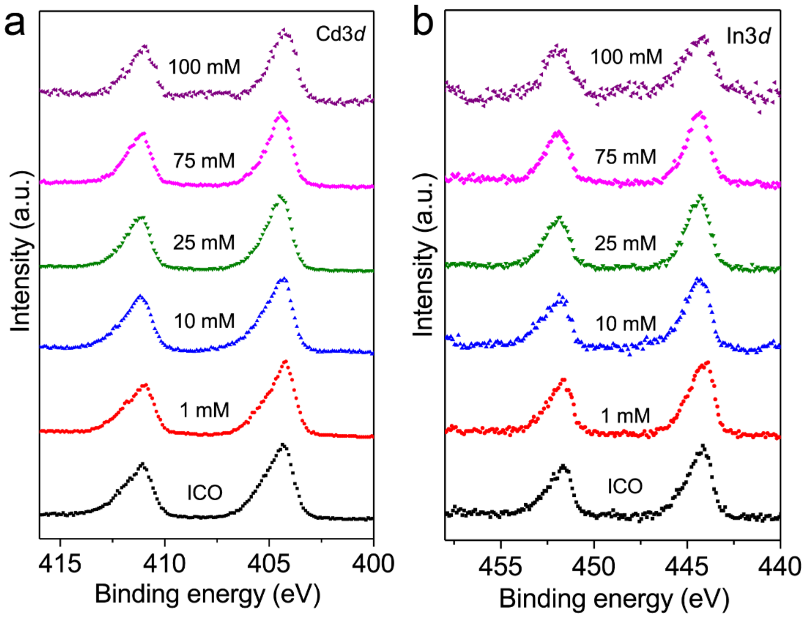


**Supplementary Figure 4 | High-resolution XPS characterization**. XPS core-level spectra in (a) Cd3*d* and (b) In3*d* regions for Cu:ICO NCs synthesized by reacting ICO NCs ($\lambda_{initial}$ = 2196 nm) with different concentrations of $CuCl_2$. These nearly identical spectra suggest that reactions with $CuCl_2$ did not alter the oxidation state of Cd or In.

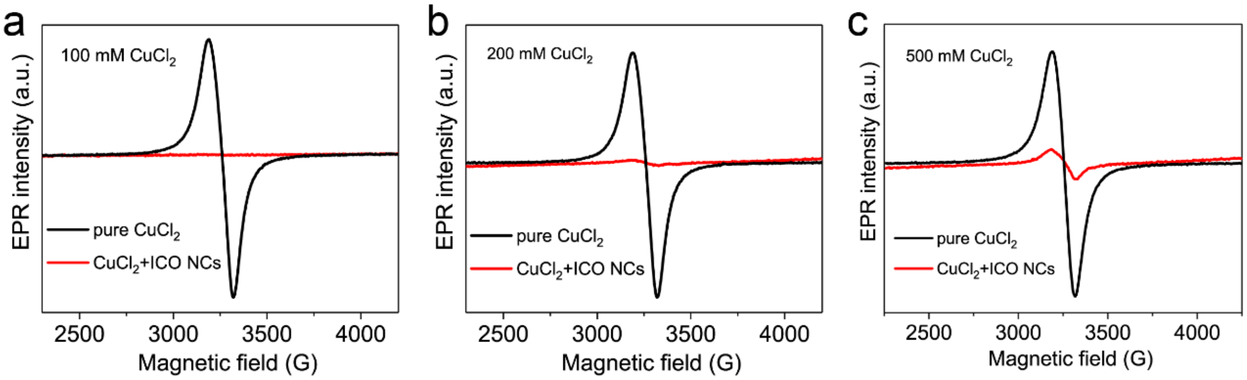


**Supplementary Figure 5 | EPR spectra of $CuCl_2$ solutions before and after reaction with ICO NCs.** (a) EPR spectra of 0.2 mL of 100 mM $CuCl_2$ solution exhibiting characteristics due to paramagnetic $Cu^{2+}$ ions. These spectral features disappeared after reacting with 10 mg of ICO NCs (dissolved in 1 mL of toluene) at 60 °C for one hour, indicative of complete reduction of $Cu^{2+}$ to $Cu^{+}$. Analogously, the EPR signal intensity diminished drastically (although not completely) after reacting the same amount of ICO NCs with 0.2 mL of (b) 200 mM and (c) 500 mM $CuCl_2$ solutions.

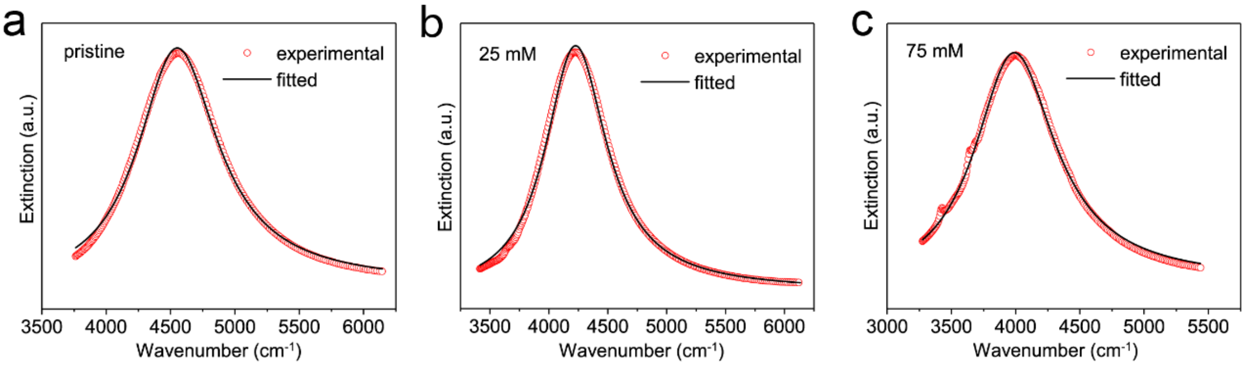


**Supplementary Figure 6 | Examples illustrating Drude fits to UV-Vis-NIR spectra for ICO and Cu:ICO NCs**.

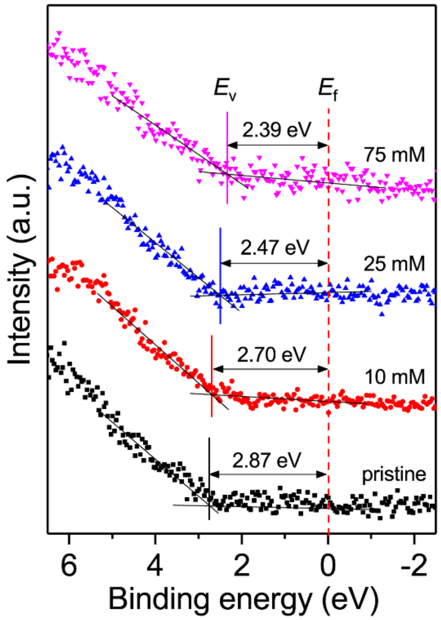


**Supplementary Figure 7 | Electronic structure characterization using UPS measurements.** UPS spectra in the valence band region for ICO NCs and Cu:ICO NCs. These data, combined with UPS spectra in the secondary electron cut-off region shown in Figure 2d, enabled deduction of the energy level diagrams shown in Figure 2e.

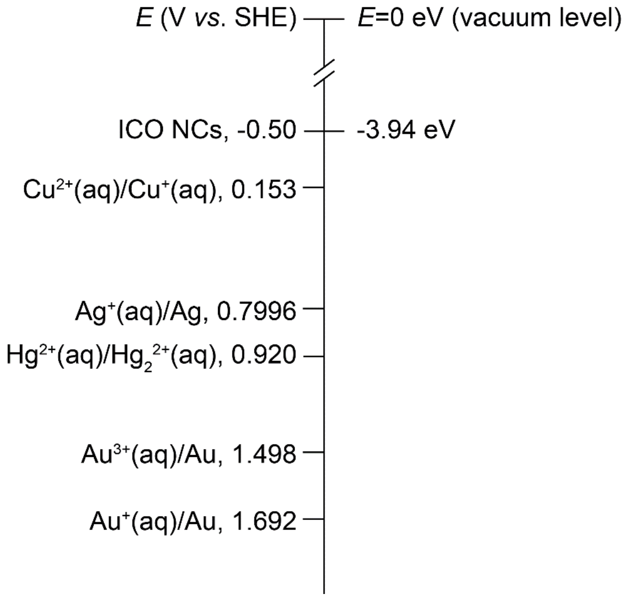


**Supplementary Figure 8 | Reduction potential of ICO NCs and selected standard reduction potentials.**[1] The Fermi energy level $E_F$ determined from UPS measurement was converted to the reduction potential (versus SHE) of ICO NCs ($\lambda_{initial}$ = 2196 nm).

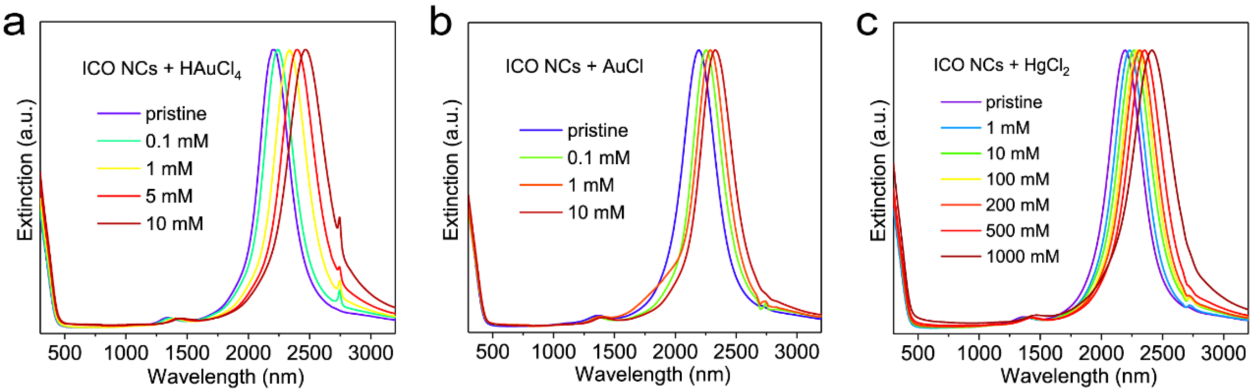


**Supplementary Figure 9 | Redox reactions between ICO NCs and gold or mercury chloride.** UV-Vis-NIR spectra of ICO NCs after reaction with different amounts of (a) $HAuCl_4$, (b) AuCl and (c) $HgCl_2$. It is found that reactions with $HAuCl_4$ or AuCl at concentrations higher than 10 mM can often lead to etching or even decomposition of ICO NCs (see for example Supplementary Figure 10e).

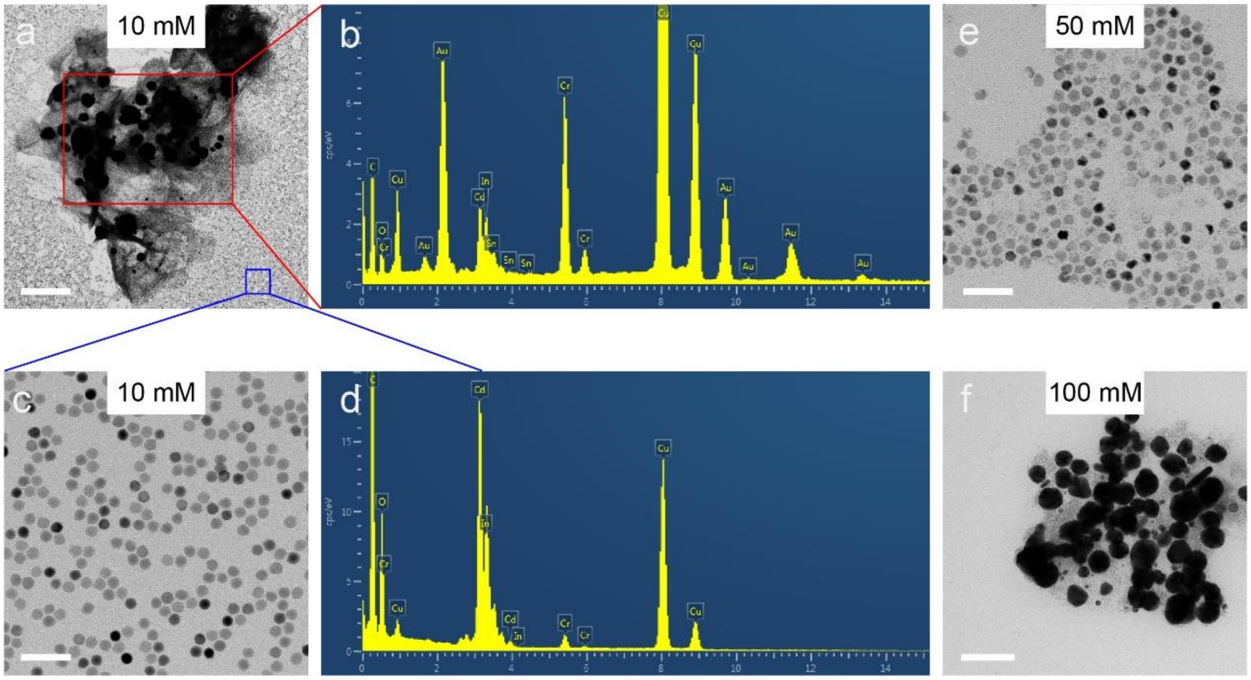


**Supplementary Figure 10 | Redox reactions between ICO NCs and $HAuCl_4$.** (a,c,e,f) TEM images of ICO NCs after reaction with different amounts of $HAuCl_4$. (b,d) TEM-EDX data acquired from the regions highlighted in (a). Scale bars: (a) 500 nm, (c,e) 50 nm, (f) 100 nm.

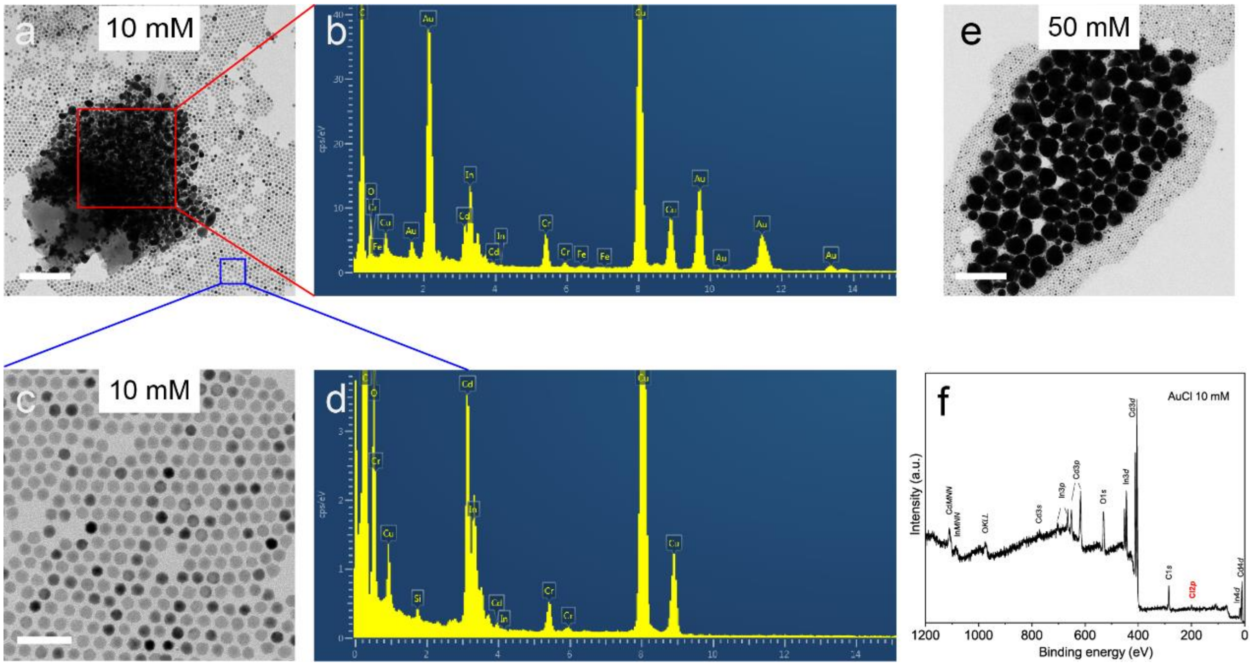


**Supplementary Figure 11 | Redox reactions between ICO NCs and AuCl.** (a,c,e) TEM images of ICO NCs after reaction with different amounts of AuCl. (b,d) TEM-EDX data acquired from the regions highlighted in (a). (f) XPS spectra for NCs shown in (c). Chlorine signal was detected for ICO NCs reacted with AuCl. Scale bars: (a,e) 200 nm, (c) 50 nm.

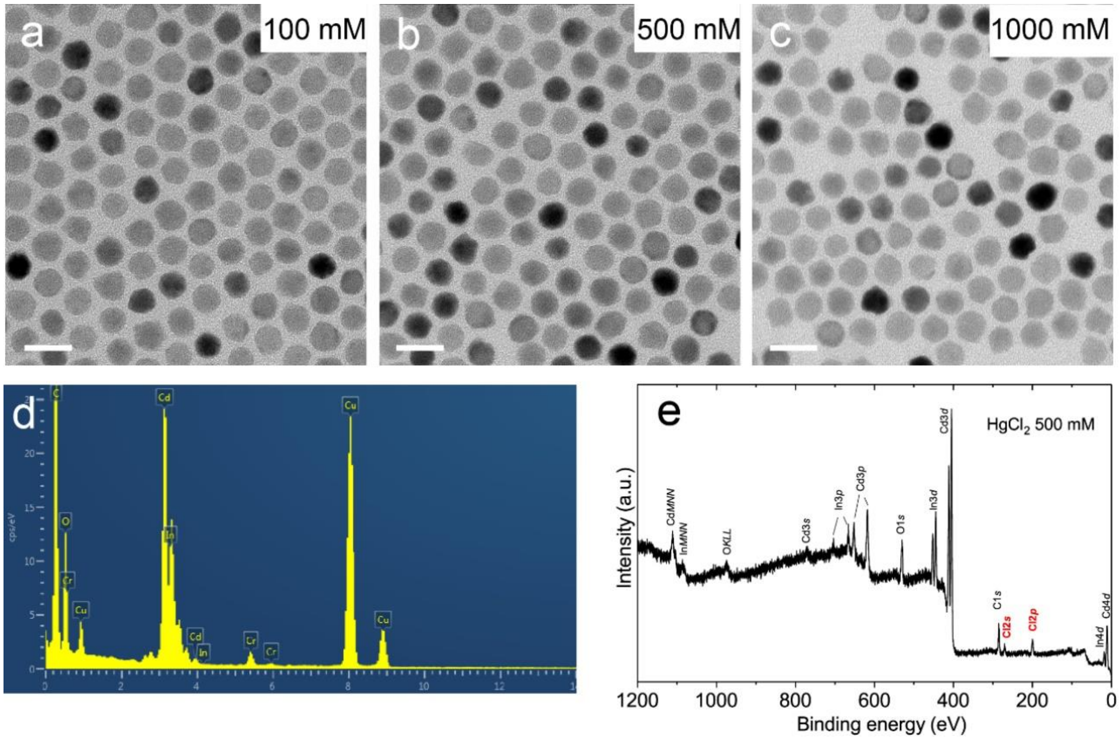


**Supplementary Figure 12 | Redox reactions between ICO NCs and $HgCl_2$.** (a-c) TEM images of ICO NCs after reaction with different amounts of $HgCl_2$. (d) TEM-EDX analysis and (e) XPS spectra of ICO NCs reacted with 0.2 mL of 500 mM $HgCl_2$. Scale bars: 20 nm.

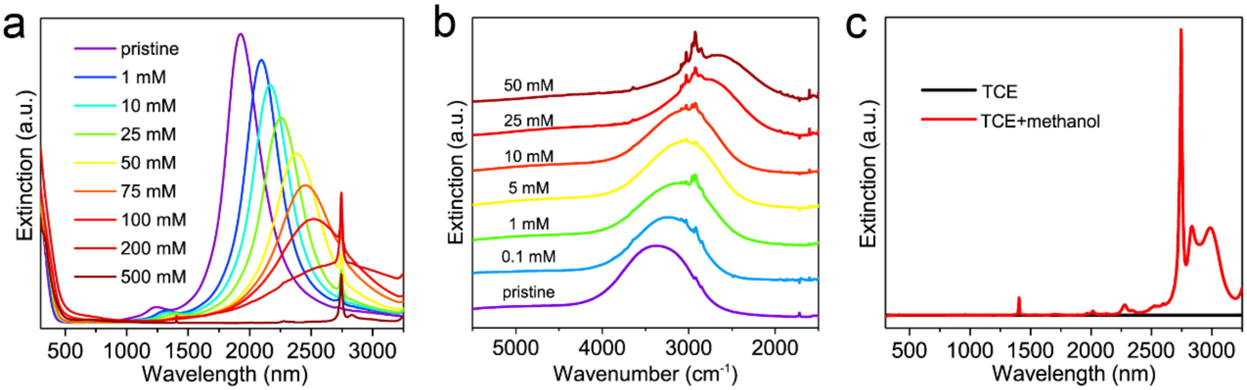


**Supplementary Figure 13 | Cation-exchange reactions between $CuCl_2$ and ICO NCs with different initial indium doping levels.** (a) UV-Vis-NIR and (b) FTIR spectra of Cu:ICO NCs synthesized by reacting (a) 16.2 % In-doped and (b) 1.1 % In-doped ICO NCs with different concentrations of $CuCl_2$. (c) UV-Vis-NIR spectra of pure TCE and methanol dissolved in TCE. The sharp peak centered at 2745 nm was also observed in the absorption spectra of many NC samples, which we have attributed to signals from residual methanol introduced during NC purification.

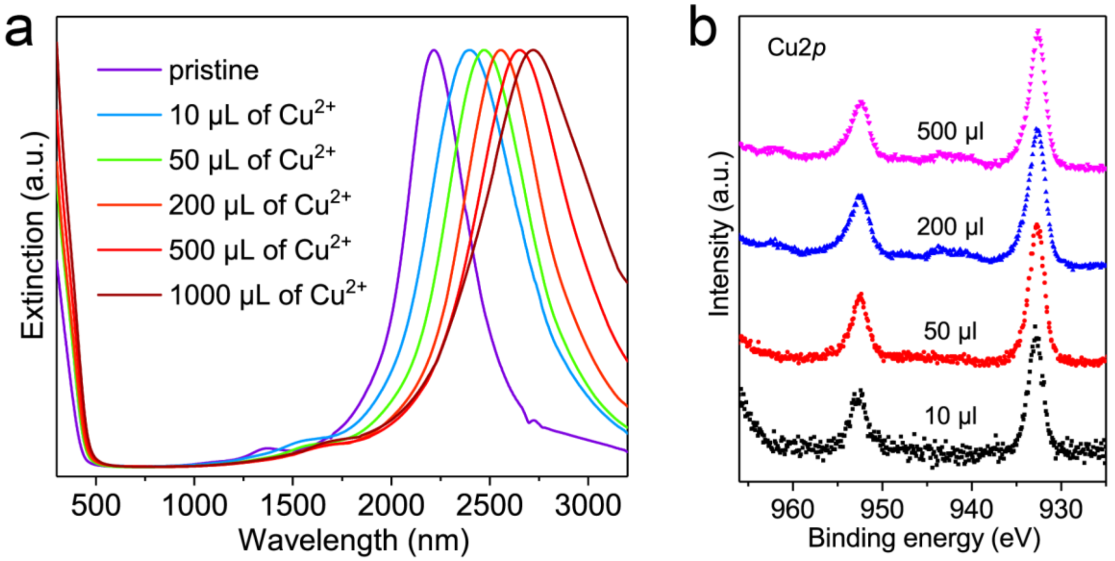


**Supplementary Figure 14 | Cation-exchange reactions between ICO NCs and DDAB-DDA-$CuCl_2$.** (a) UV-Vis-NIR spectra and (b) high-resolution XPS spectra in the Cu2*p* region for Cu:ICO NCs synthesized by reacting ICO NCs and different amounts of DDAB-DDA-$CuCl_2$.

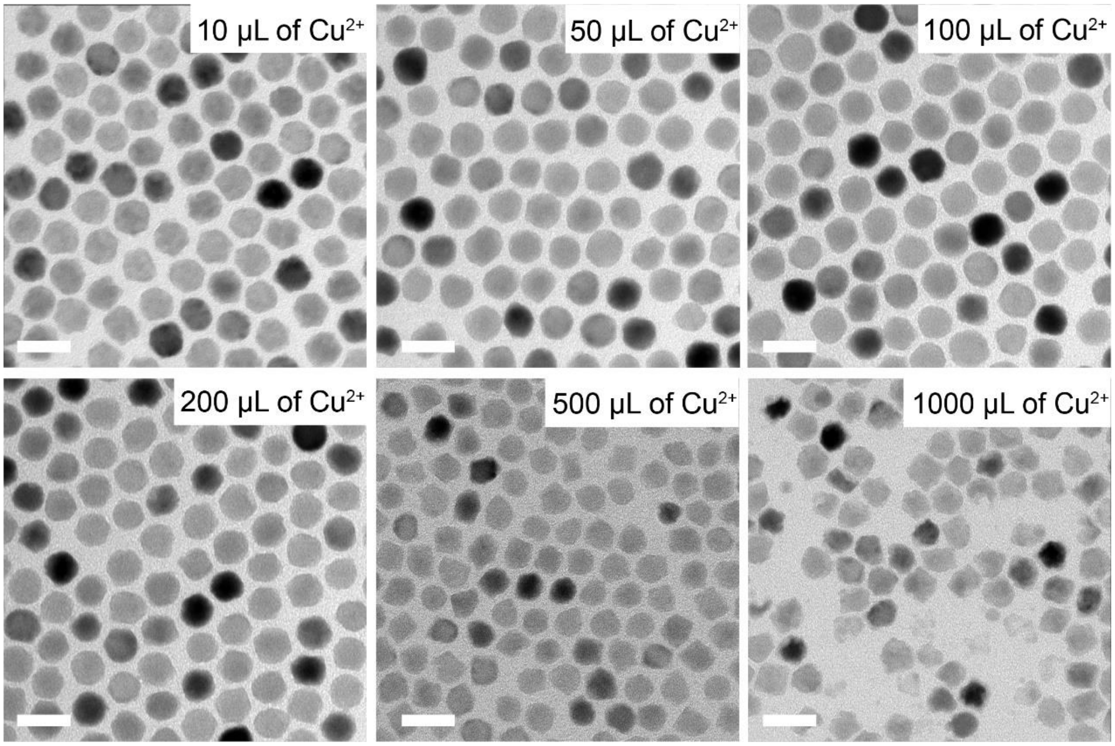


**Supplementary Figure 15 | TEM images of Cu:ICO NCs synthesized by reacting ICO NCs with different amounts of DDAB-DDA-$CuCl_2$.** Scale bars: 20 nm.

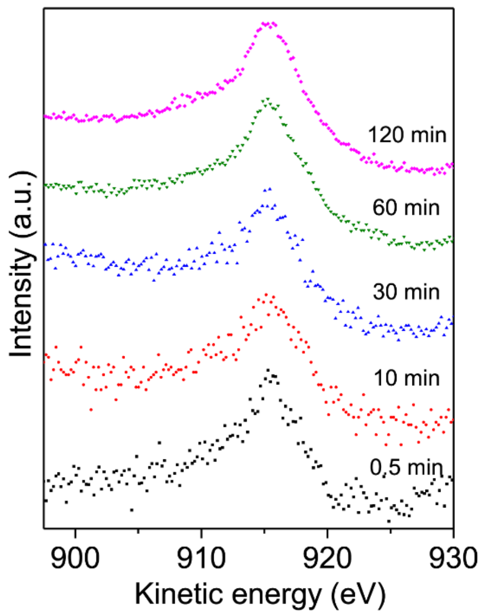


**Supplementary Figure 16 | XPS spectra in the Cu *LMM* region for Cu:ICO NCs synthesized by reacting ICO NCs with 100 mM $CuCl_2$ for different times.** The Cu *LMM* peak shifted from ~915.7 eV (0.5 min) to ~ 915.3 eV (120 min). While typical Cu *LMM* peak values for Cu (I) fall in the range of 916.5-916.9 eV,[2, 3] the *LMM* peak of CuCl and $CuCl_2$ were previously determined to be at ~915.1 eV.[3] The latter is better matched with experimentally measured values from Cu:ICO NCs. These XPS data also provide further evidence that $Cl^-$ ions were adsorbed onto NC surface upon completion of the $Cu^+/Cd^{2+}$ exchange reaction.

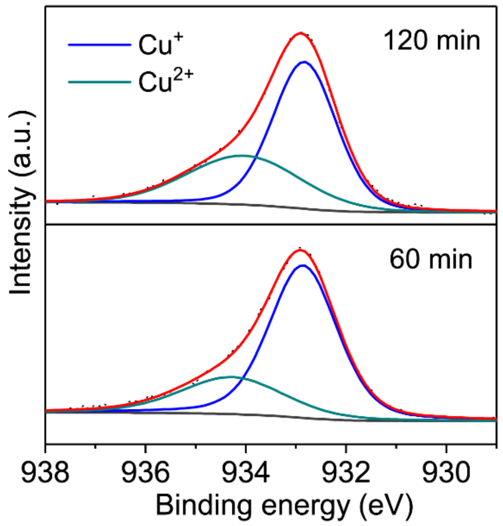


**Supplementary Figure 17 | Determination of the atomic ratio between $Cu^{+}$ and $Cu^{2+}$ for Cu:ICO NCs.** Deconvolution of the XPS spectra shown in Figure 3b for Cu:ICO NCs synthesized by reacting ICO NCs ($\lambda_{initial}$=2222 nm) with 100 mM $CuCl_2$ for 60 min and 120 min.

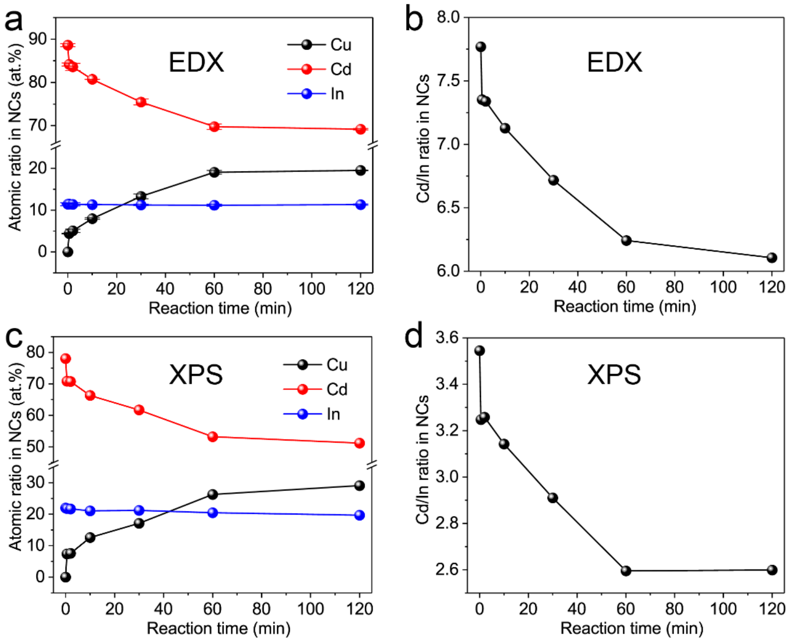


**Supplementary Figure 18 | EDX and XPS elemental analyses for Cu:ICO NCs synthesized by reacting ICO NCs ($\lambda_{initial}$=2222 nm) with 100 mM $CuCl_2$ for different times.** (b, d) Plots of calculated atom ratios between Cd and In versus reaction time based on the elemental compositions data shown in (a) and (c). The error bars in (a) represent the standard deviation between measurements on the same sample. A minimum of three SEX-EDX measurements were performed over different spots to determine the average atomic ratios presented in (a).

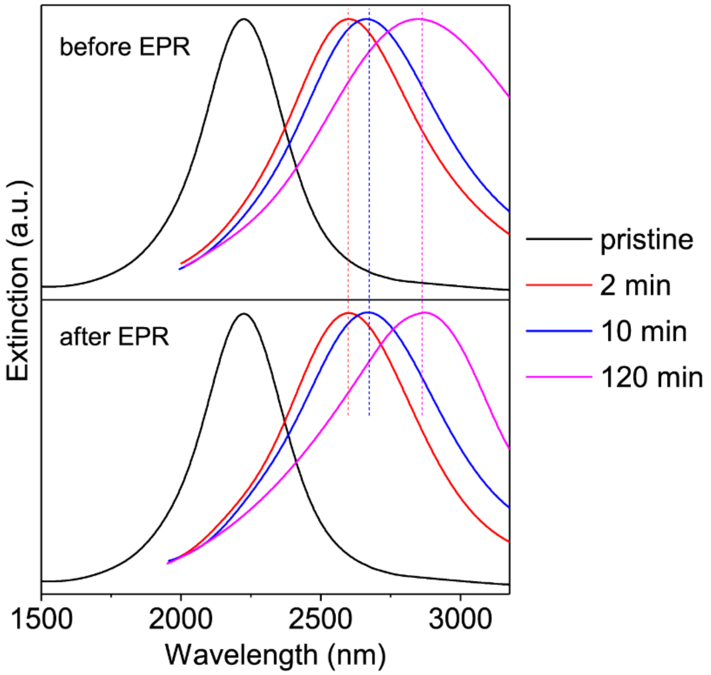


**Supplementary Figure 19 | UV-Vis-NIR spectra of NC aliquots before and after EPR measurements.** For each time point, two NC aliquots were extracted simultaneously from the reaction mixture. One aliquot was subject immediately to purification followed by acquisition of an absorption spectrum for the NCs isolated. The other aliquot was added immediately to an EPR tube, which was then rapidly frozen by plugging into liquid $N_2$. Upon completion of EPR measurements, the mixture was allowed to warm up to room temperature before NC purification. Subsequently, an absorption spectrum was recorded for NCs recovered. We found that the LSPR characteristics remained nearly identical before and after EPR measurements, which indicates that the reactions between ICO NCs and $CuCl_2$ can be successfully halted upon rapid cooling of the reaction mixture.

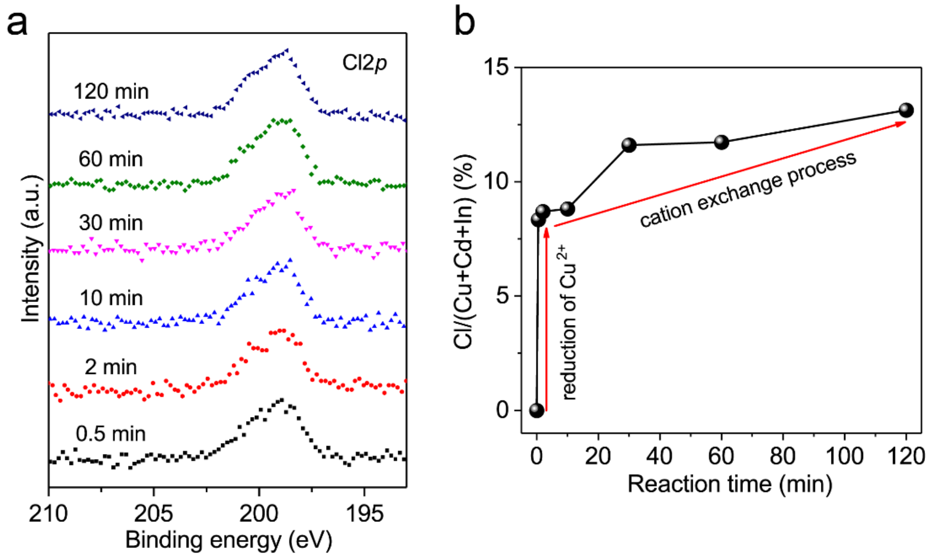


**Supplementary Figure 20 | XPS spectra in the Cl2*p* region for NCs isolated at different reaction times during the kinetic study shown in Figure 3.** (a) High-resolution XPS spectra in the Cl2*p* region for Cu:ICO NCs isolated from the reaction between ICO NCs and 100 mM $CuCl_2$ at different times. (b) Chlorine content determined by XPS for NCs isolated at different reaction times. The rapid increase in the Cl signal within the initial 30 s corresponds to the reduction of $Cu^{2+}$ by ICO NCs. The Cl concentration was found to increase gradually over the next 120 min.

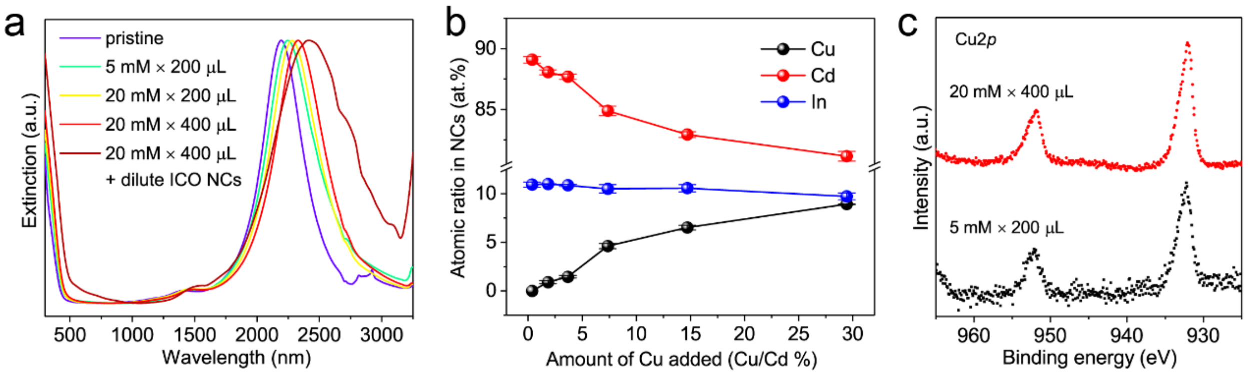


**Supplementary Figure 21 | Cation-exchange reactions using the $Cu^+$ precursor, $[Cu(CH_3CN)_4]PF_6$.** (a) UV-Vis-NIR spectra, (b) elemental analysis and (c) high-resolution XPS spectra of Cu:ICO NCs synthesized by reacting ICO NCs ($\lambda_{initial}$ = 2196 nm) with different amounts of $[Cu(CH_3CN)_4]PF_6$ dissolved in methanol. The error bars in (b) represent the standard deviation between measurements on the same sample. A minimum of three SEX-EDX measurements were performed over different spots to determine the average atomic ratios presented in (b).

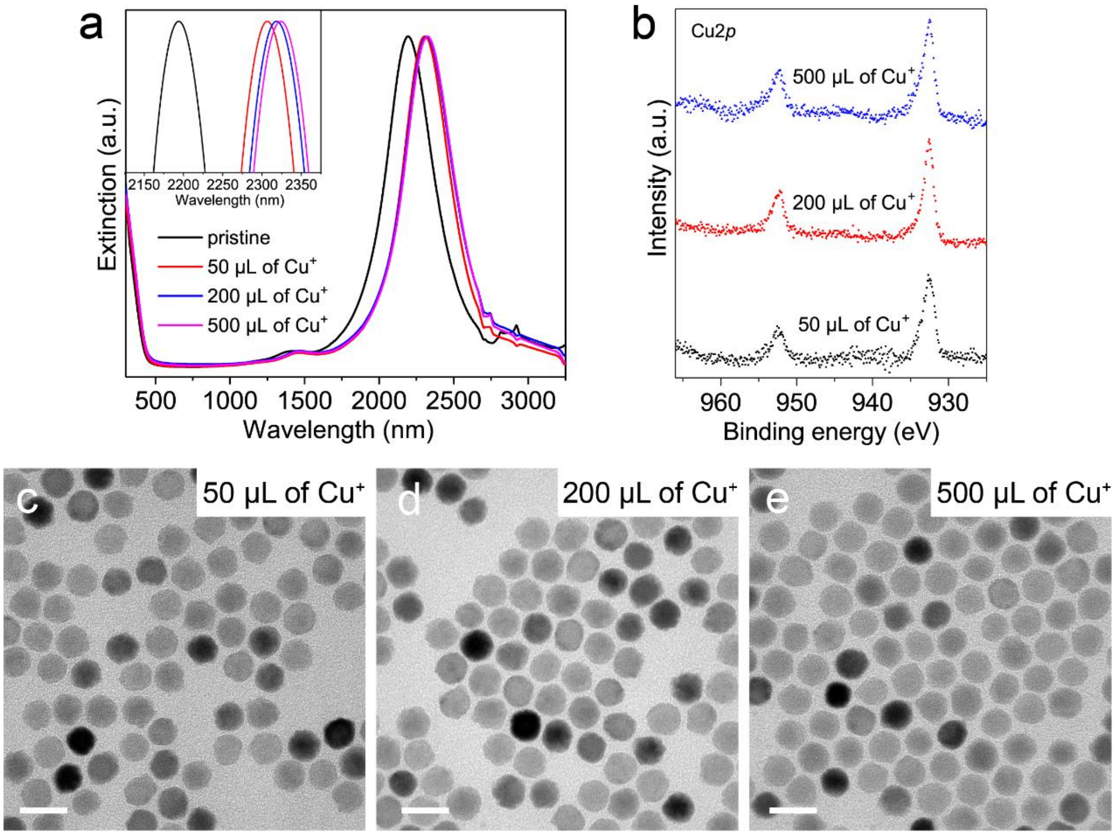


**Supplementary Figure 22 | Cation-exchange reactions using the $Cu^+$ precursor, DDAB-DDA-CuCl.** (a) UV-Vis-NIR spectra, (b) high-resolution XPS spectra and (c-e) TEM images of Cu:ICO NCs synthesized by reacting ICO NCs ($\lambda_{initial}$ = 2196 nm) with different amounts of DDAB-DDA-CuCl dissolved in toluene. Scale bars: 20 nm.

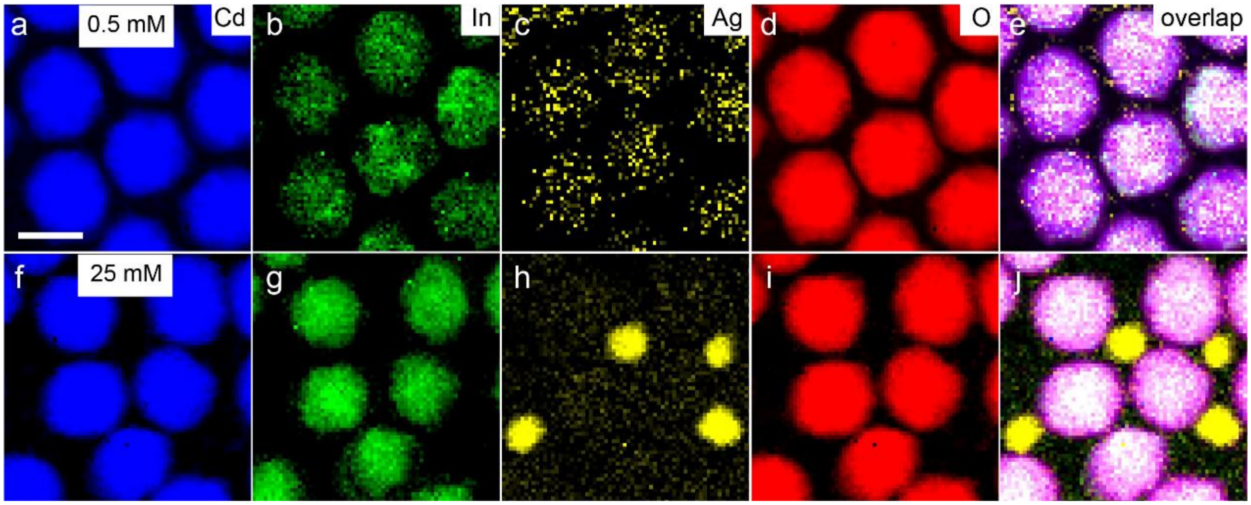


**Supplementary Figure 23 | STEM-EELS elemental mapping of Ag:ICO NCs.** (a-e) Elemental maps of Ag:ICO NCs obtained from the reaction between ICO NCs and 0.2 mL of 0.5 mM $AgNO_3$. (f-j) Elemental maps of Ag:ICO NCs resulting from the reaction between ICO NCs and 0.2 mL of 25 mM $AgNO_3$. The scale bar shown in (a) represents 10 nm and applies to all micrographs.

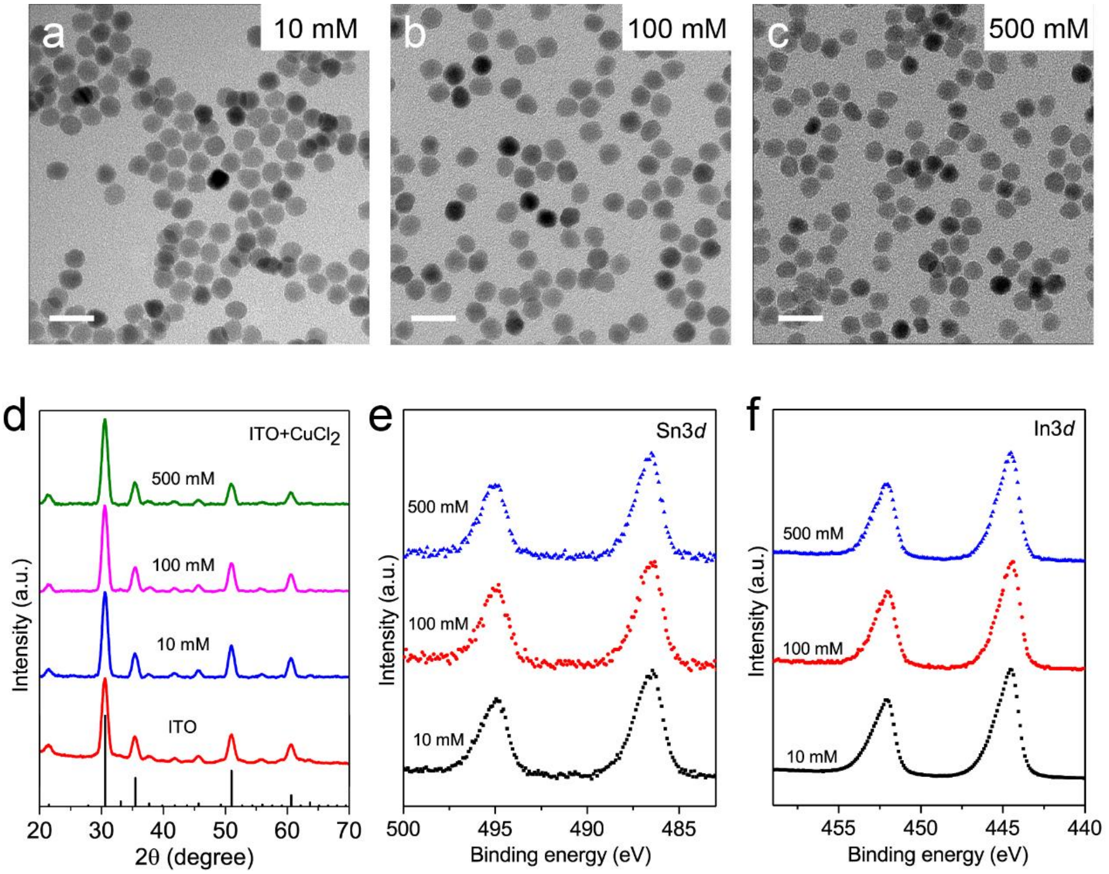


**Supplementary Figure 24 | Reactions between ITO NCs and $CuCl_2$.** (a-c) TEM images, (d) powder XRD patterns, and (e-f) high-resolution XPS spectra in the (e) Sn3*d* and (f) In3*d* regions for Cu:ITO NCs synthesized by reacting ITO NCs with different concentrations of $CuCl_2$. The pattern of vertical lines shown at the bottom of (d) corresponds to the powder XRD pattern of the cubic $In_2O_3$ phase (JCPDS Card No. 03-065-3170). Scale bars: 20 nm.

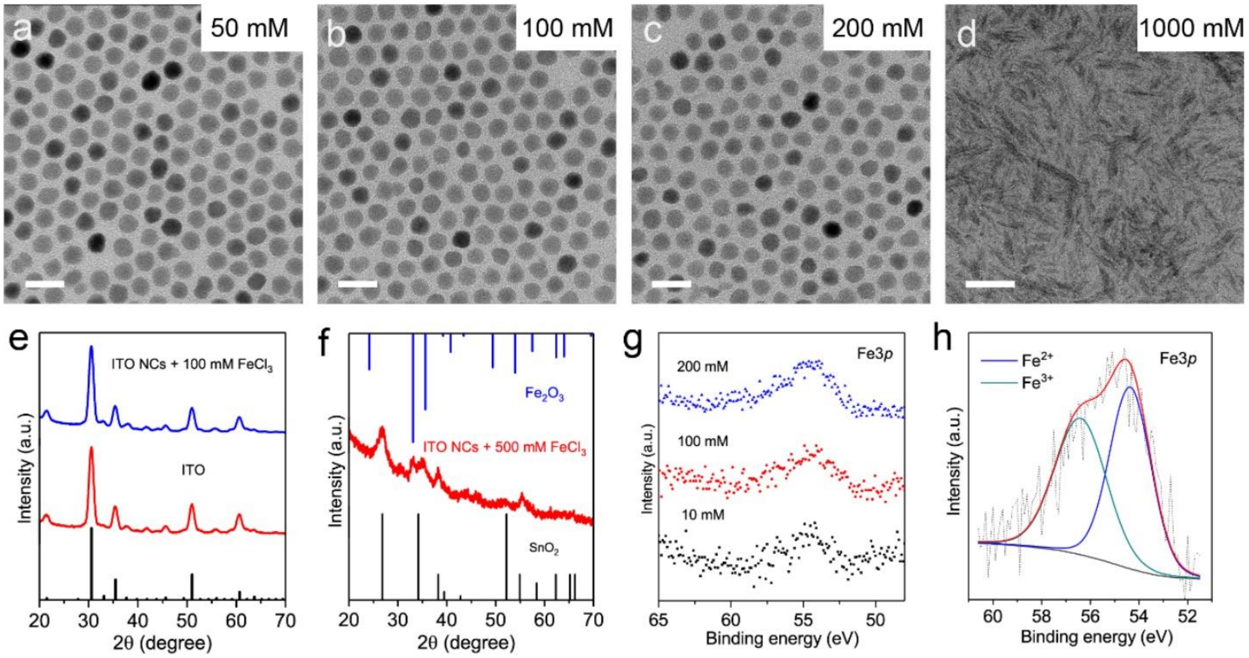


**Supplementary Figure 25 | Reactions between ITO NCs and $FeCl_3$.** (a-d) TEM images, (e-f) powder XRD patterns, and (g-h) high-resolution XPS spectra in the Fe3*p* region for Fe:ITO NCs obtained from the reaction between ITO NCs and different concentrations of $FeCl_3$. ITO NCs were consumed or destroyed when the $FeCl_3$ concentration exceeded 500 mM, and diffraction peaks attributed to $Fe_2O_3$ and $SnO_2$ phases were observed in the XRD patterns shown in (f) when such high $FeCl_3$ concentrations were used. These results can be rationalized by considering the hydrolysis of $Fe^{3+}$ and $Sn^{4+}$ ions producing corresponding metal oxides and the dissolution of ITO NCs due to strongly acidic environment. Deconvolution of the Fe3*p* XPS spectra shown in (h) for Fe:ITO NCs synthesized by reacting ITO NCs with 200 mM $FeCl_3$ reveals the presence of both $Fe^{3+}$ and $Fe^{2+}$ species within the Fe:ITO NCs. Scale bars: (a-c) 20 nm, (d) 50 nm.

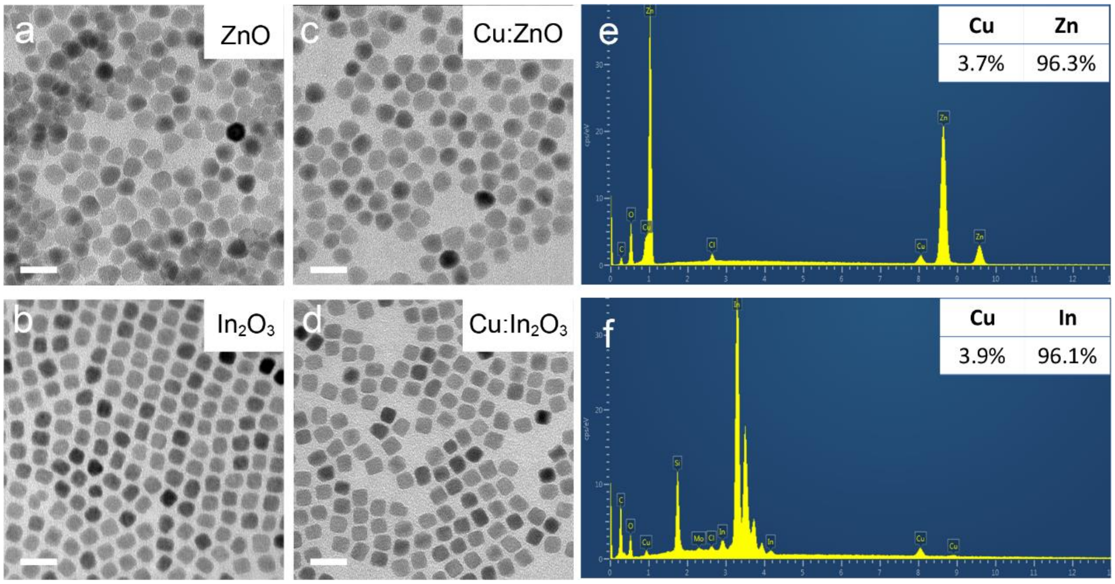


**Supplementary Figure 26 | Cation-exchange reactions using unintentionally doped metal-oxide NCs.** (a-d) TEM images of (a) ZnO, (b) Cu-exchanged ZnO, (c) $In_2O_3$ and (d) Cu-exchanged $In_2O_3$ NCs. (e,f) SEM-EDX spectra of (e) Cu-exchanged ZnO and (f) Cu-exchanged $In_2O_3$ NCs. Scale bars: 20 nm.

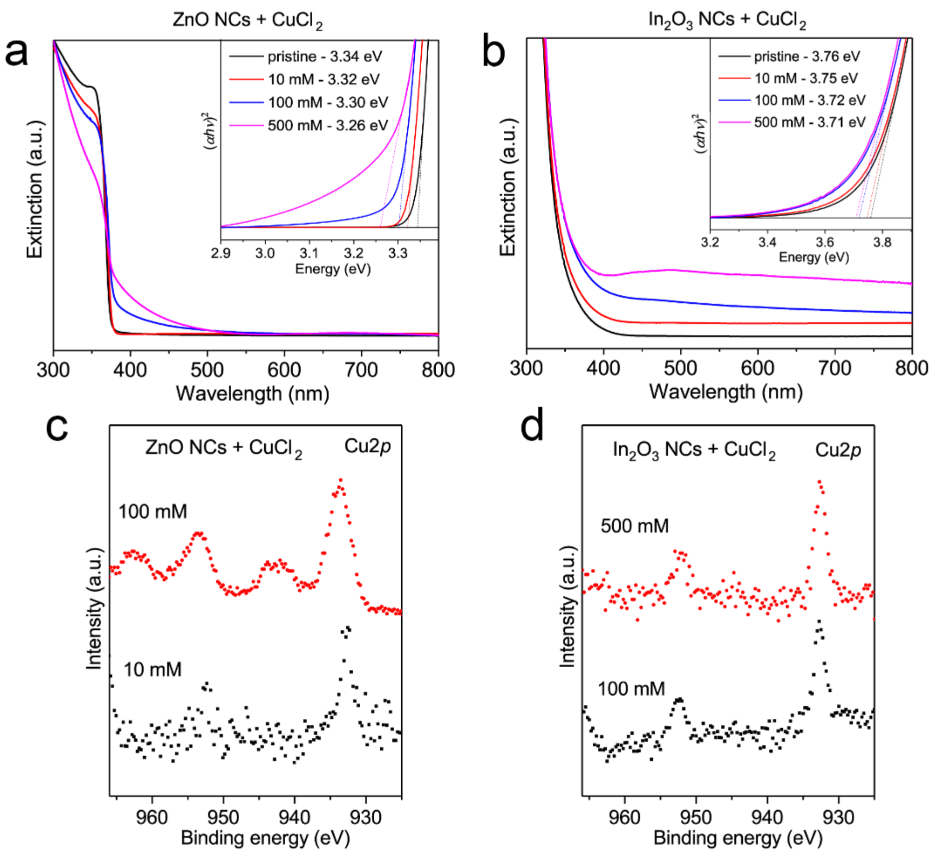


**Supplementary Figure 27 | Cation-exchange reactions between unintentionally doped metal-oxide NCs and $CuCl_2$.** (a,b) UV-Vis spectra and (c,d) XPS spectra of (a,c) Cu-exchanged ZnO NCs, and (b,d) Cu-exchanged $In_2O_3$ NCs synthesized by reacting metal-oxide NCs with different concentrations of $CuCl_2$. The inset of (a) and (b) shows plots of $(\alpha h\nu)^2$ versus photon energy. $\alpha$: absorption coefficient. The dotted lines represent extrapolation from the linear region of the curves with the x-axis intercepts indicated in the legends.

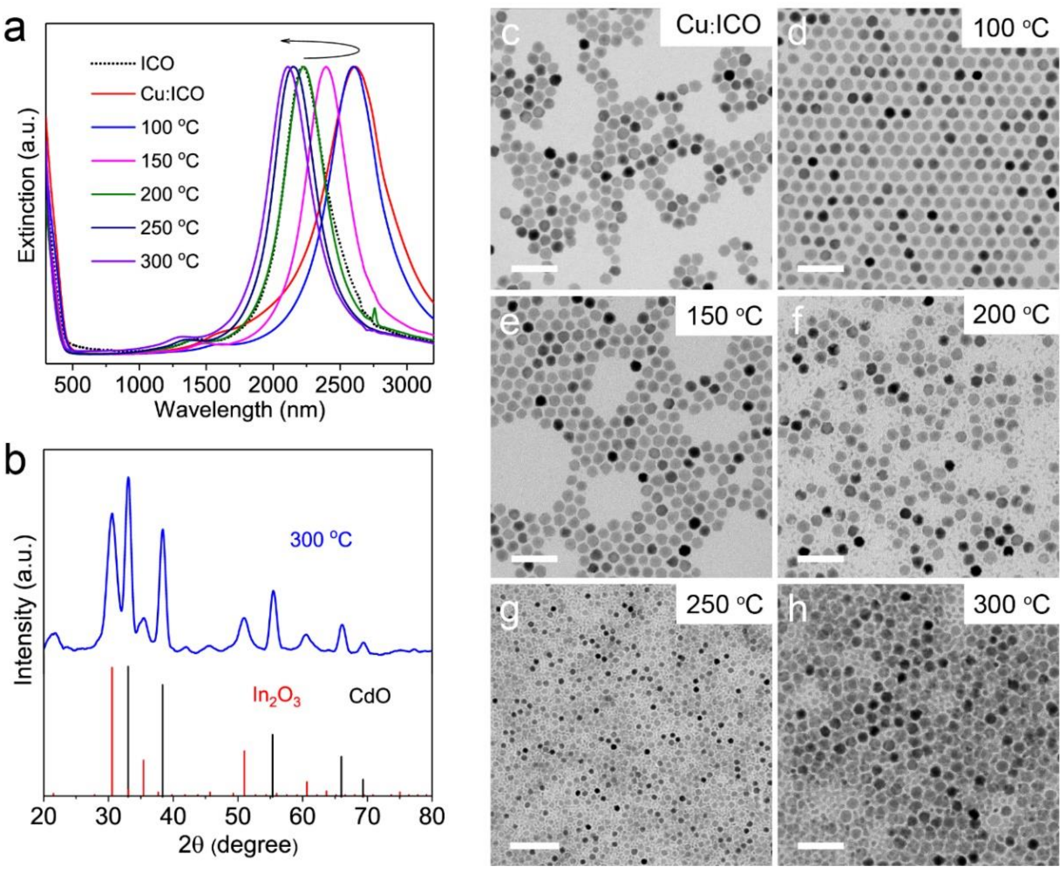


**Supplementary Figure 28 | Tuning LSPR energies of ICO NCs via sequential cation-exchange reactions.** (a) UV-Vis-NIR spectra, (b) powder XRD patterns, and (c-h) TEM images of Cu:ICO NCs and different In-exchanged Cu:ICO NCs. Scale bars: (c-f) 50 nm, (g)100 nm, (h) 200 nm. The XRD pattern in (b) indicates the formation of $In_2O_3$ NCs at elevated temperatures when ODE rather than TOPO was used as the solvent.

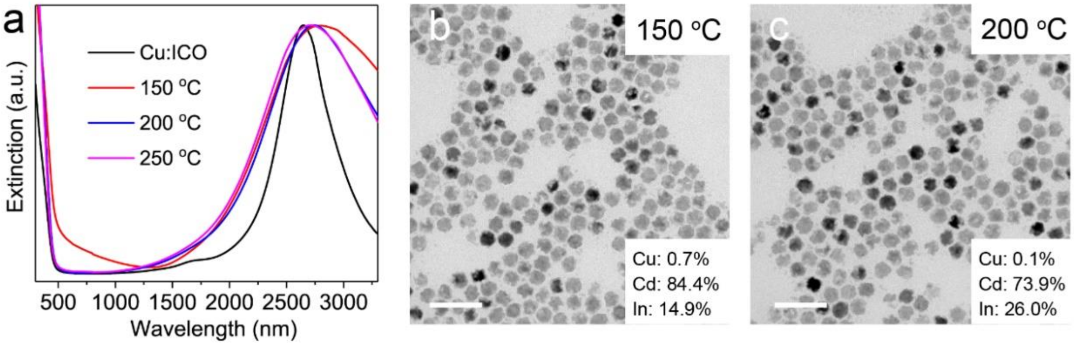


**Supplementary Figure 29 | On the critical role of TOP during cation-exchange reactions between Cu:ICO NCs and $In(ac)_3$.** (a) UV-Vis-NIR spectra and (b,c) TEM images of In-exchanged Cu:ICO NCs synthesized at different temperatures in the absence of TOP. Inset shows elemental composition ratios based on SEM-EDX analysis. Scale bars: 50 nm.

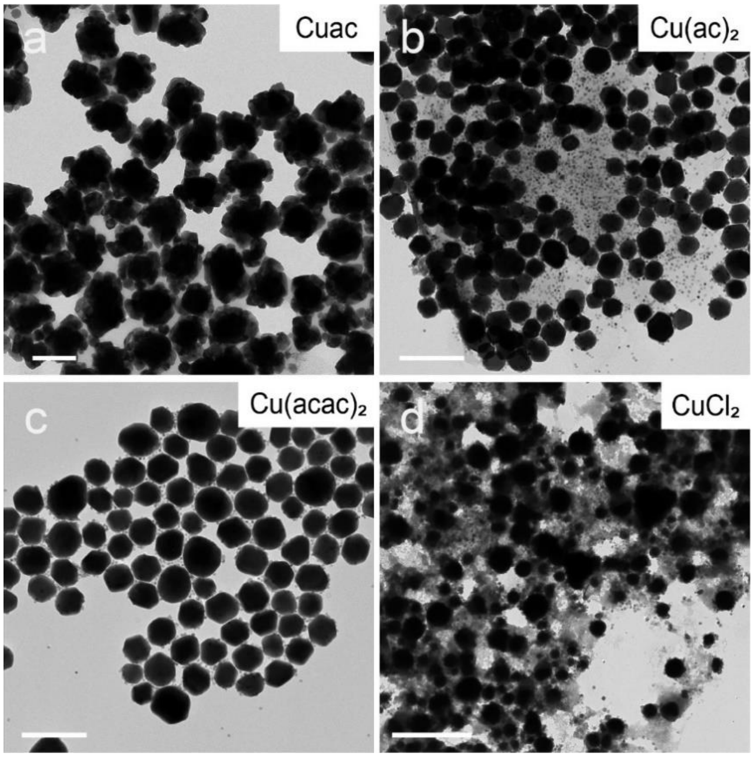


**Supplementary Figure 30 | Results from attempts to directly synthesize of Cu:ICO NCs by using the co-thermolysis method.** TEM images of NCs produced after thermal decomposition of mixed precursors composed of 80 at.% Cd(acac)$_2$, 10 at.% In(ac)$_3$ and 10 at.% (a) copper (I) acetate, (b) copper (II) acetate, (c) copper (II) acetylacetonate and (d) copper (II) chloride. Scale bars: (a-c) 200 nm, (d) 1000 nm.

## Supplementary Tables

**Supplementary Table 1 | SEM-EDX elemental analysis results for Cu:ICO NCs synthesized by reacting ICO NCs ($\lambda_{initial}$ = 2196 nm) with different concentrations of $CuCl_2$.**

| $CuCl_2$ concentration (mM) | Cu input (mmol) | Cu/Cd input (%) | Cu in NCs (%) | Cd in NCs (%) | In in NCs (%) | Cu/Cd in NCs (%) | Cd/In in NCs |
|---|---|---|---|---|---|---|---|
| 0 | - | - | - | 91.4 | 8.6 | - | 10.6 |
| 1 | 2.00E-04 | 0.4 | 0.3 | 91.1 | 8.6 | 0.3 | 10.6 |
| 10 | 0.002 | 3.7 | 1.8 | 89.6 | 8.6 | 2 | 10.4 |
| 25 | 0.005 | 9.2 | 4.2 | 87.3 | 8.5 | 4.8 | 10.3 |
| 50 | 0.01 | 18.4 | 8.2 | 83.5 | 8.3 | 9.8 | 10.1 |
| 75 | 0.015 | 27.6 | 10.9 | 81.1 | 8 | 13.4 | 10.1 |
| 100 | 0.02 | 36.8 | 14.6 | 77.6 | 7.8 | 18.8 | 9.9 |
| 200 | 0.04 | 73.6 | 33.1 | 59.7 | 7.2 | 55.4 | 8.3 |

Notes: The Cd content for initial ICO NCs was determined by using ICP-MS.

**Supplementary Table 2 | Summary of peak deconvolution results for the XPS data shown in Supplementary Figure 3.**

| $CuCl_2$ concentration (mM) | Cu species | Peak energy (eV) | Peak area (%) |
|---|---|---|---|
| 75 | $Cu^{+}$ | 932.81 | 71.3 |
| | $Cu^{2+}$ | 934.71 | 28.7 |
| 100 | $Cu^{+}$ | 932.94 | 74.4 |
| | $Cu^{2+}$ | 934.56 | 25.6 |

**Supplementary Table 3 | TEM-EDX elemental analysis results for ICO NCs reacted with $HAuCl_4$, AuCl and $HgCl_2$.**

| NC samples | Cd in NCs % | In in NCs % | Au or Hg in NCs % |
|---|---|---|---|
| ICO | 88.8±0.2 | 11.2±0.2 | - |
| ICO+10 mM $HAuCl_4$ | 88.6±0.3 | 11.1±0.3 | Au: 0.3±0.1 |
| ICO+50 mM AuCl | 88.6±0.4 | 11.2±0.4 | Au: 0.2±0.1 |
| ICO+500 mM $HgCl_2$ | 88.8±0.4 | 11.2±0.5 | Hg: 0.02±0.03 |

**Supplementary Table 4 | Summary of LSPR characteristics for Cu:ICO NCs synthesized by reacting ICO NCs ($\lambda_{initial}$ = 1928 nm) with different concentrations of $CuCl_2$.**

| $CuCl_2$ concentration (mM) | $\lambda$ (nm) | $E$ (eV) | $\Delta E$ (eV) | $Q$ | $\omega_p$ ($cm^{-1}$) | $\Gamma$ ($cm^{-1}$) | $N_e$ ($10^{20}$ $cm^{-3}$) |
|---|---|---|---|---|---|---|---|
| 0 | 1928 | 0.643 | 0.114 | 5.64 | 16330 | 851.60 | 8.05 |
| 1 | 2096 | 0.592 | 0.098 | 6.04 | 15102 | 734.33 | 6.89 |
| 10 | 2167 | 0.572 | 0.099 | 5.78 | 14611 | 744.59 | 6.45 |
| 25 | 2254 | 0.550 | 0.098 | 5.61 | 14049 | 746.40 | 5.97 |
| 50 | 2378 | 0.521 | 0.105 | 4.96 | 13315 | 788.44 | 5.36 |
| 75 | 2452 | 0.506 | 0.131 | 3.86 | 12898 | 945.27 | 5.03 |
| 100 | 2531 | 0.490 | 0.167 | 2.93 | 12531 | 1137.01 | 4.74 |
| 200 | 2751 | 0.451 | 0.259 | 1.74 | 11621 | 1666.30 | 4.08 |

**Supplementary Table 5 | Summary of peak deconvolution results for the XPS data presented in Supplementary Figure 17.**

| Reaction time (min) | Cu species | Peak energy (eV) | Peak area (%) |
|---|---|---|---|
| 60 | $Cu^{+}$ | 932.86 | 73.1 |
| | $Cu^{2+}$ | 934.27 | 26.9 |
| 120 | $Cu^{+}$ | 932.83 | 63.9 |
| | $Cu^{2+}$ | 934.04 | 36.1 |

**Supplementary Table 6 | SEM-EDX elemental analysis results for Ag:ICO NCs synthesized by reacting ICO NCs with different concentrations of $AgNO_3$.**

| $AgNO_3$ concentration (mM) | Ag in NCs % | Cd in NCs % | In in NCs % |
|---|---|---|---|
| pristine | 0 | 88.5 | 11.5 |
| 0.05 | 0.7 | 87.9 | 11.4 |
| 0.5 | 1.9 | 87.0 | 11.1 |
| 5 | 7.3 | 82.3 | 10.4 |
| 25 | 17.7 | 73.0 | 9.3 |

**Supplementary Table 7 | SEM-EDX elemental analysis results for Cu:ITO NCs synthesized by reacting ITO NCs with different concentrations of $CuCl_2$.**

| $CuCl_2$ concentration (mM) | Cu in NCs % | In in NCs % | Sn in NCs % |
|---|---|---|---|
| 0 | - | 93.5 | 6.5 |
| 50 | 2.8 | 90.8 | 6.4 |
| 100 | 3.2 | 90.1 | 6.7 |
| 500 | 5.6 | 87.8 | 6.6 |

**Supplementary Table 8 | SEM-EDX elemental analysis results for Fe:ITO NCs synthesized by reacting ITO NCs with different concentrations of $FeCl_3$.**

| $FeCl_3$ concentration (mM) | Fe in NCs % | In in NCs % | Sn in NCs % |
|---|---|---|---|
| 0 | - | 93.5 | 6.5 |
| 10 | 3.5 | 90.3 | 6.2 |
| 100 | 8.0 | 84.8 | 7.2 |
| 200 | 12.8 | 80.3 | 6.9 |
| 1000 | 96.2 | 0 | 3.8 |

**Supplementary Table 9 | Summary of LSPR characteristics and elemental analysis results for NCs undergoing sequential cation-exchange reactions.**

| | Cu (%) | Cd (%) | In (%) | $\lambda$ (nm) | $E$ (eV) | $\Delta E$ (eV) | $Q$ | $\omega_p$ ($cm^{-1}$) | $\Gamma$ ($cm^{-1}$) | $N_e$ ($10^{20}$ $cm^{-3}$) |
|---|---|---|---|---|---|---|---|---|---|---|
| ICO | 0 | 90.6 | 9.4 | 2222 | 0.558 | 0.098 | 5.69 | 14198 | 723.05 | 6.09 |
| Cu:ICO | 6.8 | 84.1 | 9.1 | 2598 | 0.477 | 0.098 | 4.87 | 12191 | 719.37 | 4.50 |
| 150 °C | 0.30 | 79.6 | 20.1 | 2196 | 0.565 | 0.096 | 5.89 | 14413 | 721.74 | 6.28 |
| 200 °C | 0 | 75.7 | 24.3 | 2086 | 0.594 | 0.103 | 5.77 | 15154 | 767.26 | 6.94 |
| 250 °C | 0 | 74.2 | 25.8 | 2010 | 0.617 | 0.110 | 5.61 | 15749 | 818.62 | 7.49 |

## Supplementary References


1. Vanysek, P. Electrochemical series. *CRC handbook of chemistry and physics* **8**, (2000).
2. Poulston, S., Parlett, P., Stone, P. & Bowker, M. Surface oxidation and reduction of CuO and $Cu_2O$ studied using XPS and XRES. *Surf. Interface Anal.* **24**, 811-820 (1996).
3. Biesinger, M. C. Advanced analysis of copper X-ray photoelectron spectra. *Surf. Interface Anal.* **49**, 1325-1334 (2017).
